# Factors Associated With Breastfeeding Outcomes in Lebanon: A National Cross‐Sectional Study

**DOI:** 10.1002/fsn3.70767

**Published:** 2025-08-06

**Authors:** Bahia Abdallah, Eman Sharara, Petra Nicolas, Hala Sacre, Cosette Fakih El Khoury, Pascale Salameh, Chadia Haddad, Joanne Karam, Rana Rizk

**Affiliations:** ^1^ Alice Ramez Chagoury School of Nursing Lebanese American University Byblos Lebanon; ^2^ Faculty of Medicine and Health Lancaster University Lancaster UK; ^3^ Department of Nutrition and Food Science, School of Arts and Sciences Lebanese American University Byblos Lebanon; ^4^ Institut National de Santé Publique, d’Épidémiologie Clinique et de Toxicologie‐Liban (INSPECT‐LB) INSPECT‐LB Beirut Lebanon; ^5^ Gilbert and Rose‐Marie Chagoury School of Medicine Lebanese American University Beirut Lebanon; ^6^ Faculty of Pharmacy Lebanese University Lebanon; ^7^ Department of Primary Care and Population Health University of Nicosia Medical School Nicosia Cyprus; ^8^ Research Department Psychiatric Hospital of the Cross Jal el Dib Lebanon; ^9^ Faculty of Public Health Lebanese University Fanar Lebanon

**Keywords:** breastfeeding, health personnel, hospitals, lactation, Lebanon, surveys and questionnaires

## Abstract

The overarching study introduces a series of papers that aim to examine the effects of these policies on breastfeeding practices; this paper presents the overall methodology and sample characteristics. It also presents percentages of breastfeeding practices and associated factors, with a particular focus on lactation support. This cross‐sectional study was conducted among a sample of 280 Lebanese adult mothers with at least one child less than 5 years old using an online survey. Descriptive and inferential statistics were carried out, where chi‐square and Fisher's exact test were used as applicable. The bivariate and multivariate logistic regressions were performed. Sociodemographic, economic, lifestyle, and pregnancy‐related variables poorly predicted breastfeeding practices, while receiving prenatal education about breastfeeding before pregnancy (OR = 1.81 [1.03–3.15]) and during the third trimester (OR = 1.71 [1.03–2.81]) was associated with higher odds of early initiation of BF. The main sources of prenatal education were gynecologists, lactation specialists, and midwives who had significant positive influences on some BF practices. The multivariate analysis showed a positive association between normal delivery with skin‐to‐skin contact (AOR = 6.08 [3.30–11.21]) and early initiation of BF (AOR = 4.07 [2.24–7.41]), whereas support from experienced mothers, receiving prenatal education about breastfeeding from lactation specialists, and support from the workplace were negatively associated with some BF practices. These findings highlight the critical need to expand breastfeeding initiatives across Lebanon, mainly by empowering healthcare professionals with enhanced training and resources.

## Graphical abstract

The four outcomes that had significant predictors in the multivariate analysis were skin‐to‐skin contact (SSC), early initiation of breastfeeding, exclusive breastfeeding, and breastfeeding continuation to 1 year. Accounting for the effect of covariates, vaginal normal delivery was significantly positively associated with SSC and early initiation of breastfeeding. Support from lactation specialists was a positive predictor of exclusive breastfeeding, whereas giving birth in 2021 and knowledge about the Ministry of Public Health's hotline were predictors of breastfeeding continuation to 1 year.

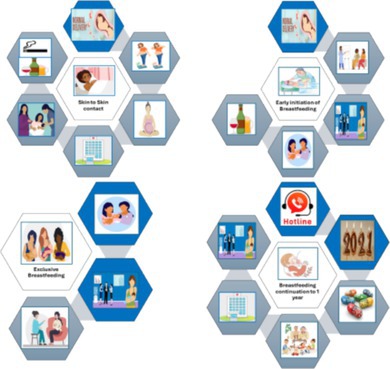

## 1 Introduction

Breastfeeding (BF), as defined by the World Health Organization (WHO), involves feeding infants human breast milk, either directly from the breast or via expressed milk, to provide optimal nutrition during early life (WHO 2023a, 2023b). The WHO recommends exclusive BF for the first 6 months of an infant's life, followed by continued BF with appropriate complementary foods for up to 2 years or beyond (WHO 2023a, 2023b). Optimal BF practices enhance infant growth, health, and development (Victora et al. 2016), where breast milk uniquely supplies the nutritional needs of infants, offering a composition that adjusts over time to meet their growing demands (Couto et al. 2020). Breast milk contains essential antibodies that protect against low‐risk common childhood illnesses such as ear infections and gastroenteritis or even serious ones such as diarrhea and respiratory infections, significantly reducing infant mortality rates (Couto et al. 2020; Lackey et al. 2021). Beyond nutrition, BF enhances cognitive development and intelligence scores in later childhood (Horta et al. 2015) and provides significant benefits to the mother, such as reduced risk of breast and ovarian cancers, faster postpartum weight loss, and decreased likelihood of postpartum depression (Ahmadinezhad et al. 2024; Eoh et al. 2021; He et al. 2015). Economically, BF offers substantial cost savings by reducing the need for medical care and infant formula (Rollins et al. 2016).

Despite the numerous benefits of BF, there has been a notable decline in its practice worldwide, with the WHO reporting that less than 40% of infants globally are exclusively breastfed during the first 6 months of life (WHO 2023b). This decline is mirrored across the Middle East and North African (MENA) region, where exclusive BF rates are particularly low. For example, a study in 2021 found that only around 28% of mothers practiced exclusive BF for the first 6 months in Saudi Arabia (Alyousefi 2021). In addition, in Qatar, a study in 2017 found that while 96.2% of mothers initiate BF, only 24.3% continue to exclusively breastfeed by the sixth month, indicating a significant drop‐off as infants age (Hendaus et al. 2018). This shift is primarily due to cultural preferences and strong formula marketing (Hendaus et al. 2018). These rates are alarmingly lower than the WHO's recommended target of 50% (WHO 2023a).

The trends in Lebanon are also concerning. Recent data have shown that the prevalence of exclusive BF up to 6 months postpartum is 37.7% (Zablith and Reilly 2020). Similarly, a low rate of exclusive BF for the first 6 months (22.2%) was presented by the most recent national nutrition survey conducted by the United Nations Children's Fund (UNICEF) and the Ministry of Public Health (MoPH) in collaboration with Action Against Hunger Canada SMART team from July to September 2021 (Lebanon Nutrition Sector 2022). Factors influencing this rate include socio‐cultural‐demographic and health system‐related factors such as maternal employment, whether the pregnancy was planned, the mother's intention to breastfeed, the source of maternal emotional support, and access to postpartum support services, such as a hotline or a support film (Hamade et al. 2013; Zablith and Reilly 2020). While the initiation rate of BF in Lebanon reached 96% in 2020, the exclusive BF rates for infants under 6 months were found to drop significantly to around 15% (Oueidat et al. 2020). This sharp decline highlights the urgent need for interventions such as uninterrupted skin‐to‐skin contact (SSC) and early initiation of BF, which are crucial interventions supported by the WHO to improve BF rates (WHO 2023a).

The challenges to maintaining BF in Lebanon include various cultural and socioeconomic factors, in addition to the lack of widespread implementation of supportive practices such as SSC and professional BF support in hospitals (Oueidat et al. 2020). To address these challenges, Lebanon has implemented several strategic initiatives to support BF mothers. These include the implementation of BF policy frameworks and the Baby‐Friendly Hospital Initiative (BFHI), designed to bolster hospital practices to better support BF mothers (Hamade et al. 2013). Additionally, a national BF hotline and awareness campaigns were established to enhance community outreach and counseling services, effectively extending support to BF mothers. The UNICEF's Nutrition in Times of Crisis report outlines these efforts, noting significant outreach through social media that reached approximately 4.2 million people in 2021, thus improving public awareness and understanding of optimal Infant and Young Child Feeding (IYCF) practices (UNICEF 2022). Complementary to these is the strategic framework outlined in the UNICEF Nutrition Strategy for the MENA region 2020–2030 (UNICEF 2020) and the Regional Nutrition Situation Analysis (UNICEF 2024), which stresses the importance of integrating nutrition strategies into national health and development agendas. Collectively, these initiatives reflect Lebanon's commitment to enhancing maternal and child health through improved BF practices: SSC, early initiation of BF, exclusive BF for 6 months, continued BF to 1 year, and continued BF to 2 years, as part of a broader strategy to advance public health and nutrition.

## 2 Aim

This paper introduces the methodology of this study, where a series of articles will be consecutively published, and examines the factors associated with BF practices in Lebanon.

The specific objectives of this paper are to explore BF practices among mothers with children less than 5 years old using online‐based survey data, and to examine the general factors associated with BF practices, with special emphasis on the effect of lactation support from different healthcare providers (HCPs) on BF practices.

## 3 Methodology

### 3.1 Design

This cross‐sectional study was conducted among 280 Lebanese adult mothers using an online survey on Google Forms.

### 3.2 Participants

Women aged 18 years and older, residing in Lebanon but not in refugee camps, who have given birth at any time between 2018 and 2023, including those who gave birth outside Lebanon but live in Lebanon, and who are willing to complete the online survey, were recruited via social media platforms, including targeted Facebook and WhatsApp groups. Exclusion criteria included women residing in refugee camps or Lebanese women residing outside of Lebanon, and those unable to complete the online survey due to technical limitations or other reasons.

### 3.3 Sample Size

The sample size was determined based on the overall aims of the study, where multiple regressions and factor analyses will be conducted. Thus, a minimum sample of 300 participants was targeted, assuming a small effect size (f2 = 0.11), expecting a squared multiple correlation (R2) of 0.1, and considering an alpha error of 5%, a power of 80%, and allowing the inclusion of 20 predictors in the model.

### 3.4 Data Collection

Data collection occurred between early December 2023 and mid‐March 2024. The questionnaire was available in English and Arabic, where participants could choose their preferred language. The questionnaire included the following sections:

1. Sociodemographic, lifestyle, and employment questionnaire: included questions about age, marital status, number of children, educational level, place of residence, area of living, monthly household income, smoking habits, alcohol consumption, intake of dietary supplements, and current occupation.
2. Pregnancy questionnaire: included questions about self‐reported anthropometric data (height, prepregnancy weight, gestational weight gain), in addition to the mode of delivery and number of weeks of pregnancy.
3. BF practices questionnaire: included questions about self‐reported SSC contact immediately after birth, self‐reported early initiation of BF within the first hour after birth, self‐reported exclusive BF at 6 months, and self‐reported BF continuation at 1 year and 2 years.
4. The WHO and UNICEF Ten Steps to Successful Breastfeeding (WHO 2018)
5. Breastfeeding Behavior Questionnaire (BBQ) (Libbus 1992).
6. Patient Health Questionnaire (PHQ‐4): this 4‐item validated tool was used to assess psychological distress (Christodoulaki et al. 2022; Kroenke et al. 2009; Löwe et al. 2010).
7. Women's Health Initiative Insomnia Rating Scale (WHIIRS) (Levine et al. 2003).
8. Mediterranean Diet Adherence Screener (MEDAS): (Bekar and Goktas 2023; García‐Conesa et al. 2020).
9. BF Food Security Assessment Scale (BFSAS): included selected questions from the 21‐item Household Food Insecurity Experience Scale (HFIES) (Cafiero et al. 2018) and the 9‐item Household Food Insecurity Access Scale (HFIAS) (Coates et al. 2007).

To ensure valid and reliable results, the aforementioned were used. Some of these tools were tested among the Lebanese, in Arab countries, or in other contexts, for instance: BBQ (Ali et al. 2014; Bajoulvand et al. 2019; Charafeddine et al. 2020; Hamade et al. 2014), PHQ‐4 (Löwe et al. 2010; Obeid et al. 2024; Stanhope 2016; Wicke et al. 2022), and MEDAS (García‐Conesa et al. 2020; Hashim et al. 2024; Hebestreit et al. 2017; Sammoud et al. 2023; Schröder et al. 2021).

### 3.5 Data Analysis

Data were analyzed by outcomes using STATA software version 18: (1) SSC; (2) early initiation of BF; (3) exclusive BF until 6 months; (4) continued BF to 1 year; and (5) continued BF to 2 years. Some variables were calculated based on the information presented, such as the body mass index (BMI) and pregnancy period (preterm or full‐term). Both descriptive and bivariate inferential statistics were carried out, where the chi‐square test, Fisher's exact test, and simple logistic regression were used as applicable. The multiple logistic regression was performed to account for intervening variables, where only significant variables (*p* value < 0.05) in the bivariate analysis were included in the final models.

## 4 Results

### 4.1 Sociodemographic, Economic, and Health Characteristics

A total of 280 mothers with at least one child less than 5 years old were included in the study, with no missing data available due to the online format with the requirement feature. Participants ranged between 18 and 49 years, with a mean age of 31.8 years; they had between 1 and 5 children, of whom 1 to 3 were under 5 years. More than half of the participants gave birth in 2022 (24.7%) and 2023 (35.3%). Almost all (99.3%) were married, while only two respondents were divorced. Almost half (46.1%) were postgraduates, 40% were bachelor‐level university graduates, and only 3.6% were of intermediate educational level or less (Table 1). Most of the respondents resided in Mount Lebanon (43.9%) and Beirut (24.3%) and had either three or fewer (45.7%) or four (35.2%) family members in the household, with 38.2% having a monthly household income of more than 2000 US$ (Table 1). They had various occupations in the scientific/health (18.6%) and nonscientific (32.1%) fields or were housewives (20.7%); few (21.1%) stopped working to take care of their babies (Table 1).

**TABLE 1:** Descriptive statistics for sociodemographic and economic characteristics of participants (*n* = 280).

	*N* (%)
Marital status	
Divorced	2 (0.7)
Married	278 (99.3)
Educational level	
Intermediate or less	10 (3.6)
Secondary and technical	29 (10.3)
University	112 (40.0)
Postgraduate	129 (46.1)
Residence (By governorate)	
Beirut	68 (24.3)
Beqaa	25 (8.9)
Mount Lebanon	123 (43.9)
North	22 (7.9)
South	42 (15.0)
Number of family members live in the household	
<=3	127 (45.7)
4	98 (35.2)
5	38 (13.7)
> = 6	15 (5.4)
Number of rooms in the household	
<=2	21 (7.5)
3	69 (24.6)
4	92 (32.9)
5	68 (24.3)
> = 6	30 (10.7)
Income	
No income—< 450$	65 (23.2)
450$–999$	47 (16.8)
1000$–2000$	61 (21.8)
> 2000$	107 (38.2)
Occupation	
Housewife	52 (18.6)
Employed/Freelancer/Contractor in a scientific or health field	90 (32.1)
Employed/Freelancer/Contractor in another field	58 (20.7)
Stopping work to take care of child	59 (21.1)
Self‐employed in a scientific or health field	17 (6.1)
Student/Trainee	4 (1.4)

While the majority were noncigarette smokers (82.5%) and nonalcohol consumers (73.6%), slightly more than half were nonwaterpipe smokers (57.9%), and 15% were previous smokers. Participants' consumption of dietary supplements varied greatly, with the highest used being vitamin D (49.3%), followed by multivitamins (36.1%) (data not shown).

The vast majority (95.4%) of the participants delivered in private hospitals. While slightly more than half (57.2%) had a cesarean section with local anesthesia, only 9.6% had a normal vaginal delivery without local anesthesia, and almost one‐third (29.4%) had a preterm baby (Table 2).

**TABLE 2:** Descriptive statistics of delivery‐related characteristics of participants (*n* = 280).

	*N* (%)
Year of last birth	
2018	8 (2.9)
2019	15 (5.4)
2020	30 (10.9)
2021	51 (18.6)
2022	68 (24.7)
2023	97 (35.3)
2024	6 (2.2)
Delivery place	
Public hospital in Lebanon	5 (1.8)
Private hospital in Lebanon	276 (95.4)
Home birth	2 (0.7)
Hospital outside Lebanon	6 (2.1)
Mode of delivery	
Cesarean section with/without general anesthesia	163 (58.2)
Normal Vaginal delivery with/without local anesthesia	117 (41.8)
Pregnancy time period	
Preterm	80 (29.4)
Full‐term	192 (70.6)

### 4.2 Prevalence of BF Practices

**FIGURE 1:**
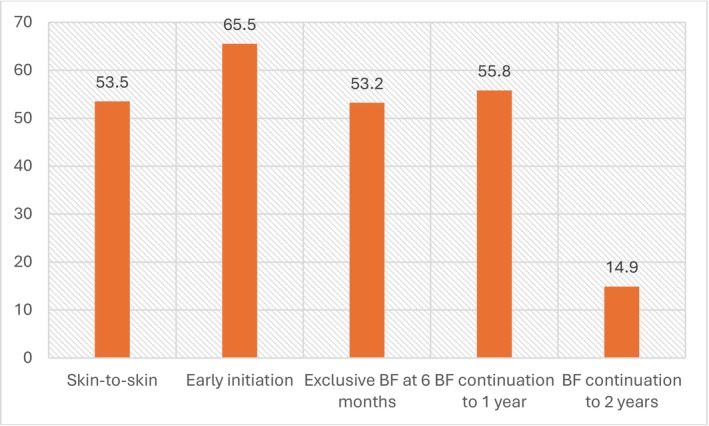
Prevalence of breastfeeding practices (*n* = 280).

Most participants (65.5%; *n* = 182) reported an early initiation of BF (within an hour after delivery), yet only 53.5% (*n* = 147) had SSC. Almost half of the mothers (53.2%; *n* = 149) exclusively breastfed until 6 months, 55.8% (*n* = 139) continued BF to 1 year, and only 14.9% (*n* = 37) continued to 2 years (Figure 1).

### 4.3 Associations Between Sociodemographic, Economic, and Health Characteristics and BF Practices

The bivariate analysis showed a few significant associations between the sociodemographic, economic, and health characteristics and the five outcomes, that is, SSC, early initiation of BF, exclusive BF until 6 months, continued BF to 1 year, and continued BF to 2 years (Supporting Information 1). BMI values before (OR = 0.91; 95% CI = 0.85–0.96) and during (OR = 0.92; 95% CI = 0.87–0.97) the last month of pregnancy were significantly higher among mothers undergoing SSC (*p* = 0.001 and *p* = 0.002, respectively). Income was significantly associated with SSC, with more affluent women being more likely to experience SSC (*p* = 0.001). Although deliveries in public hospitals were limited (*n* = 5), none of the mothers had SSC. In contrast, half of the deliveries in private hospitals and four of five deliveries outside Lebanon involved SSC (*p* = 0.031). The mode of delivery was significantly associated with SSC; women who had a normal delivery, with or without anesthesia, were more likely to have SSC compared to those who underwent cesarean sections (76.9% vs. 36.1%, OR = 5.91; 95% CI = 3.45–10.12).

The only variable significantly associated with the early initiation of BF was the mode of delivery. Women who had normal deliveries, with or without anesthesia, were more likely to initiate BF earlier compared to those who had cesarean sections (82.9% vs. 52.8%, OR = 4.34; 95% CI = 2.45–7.68).

BF continuation to 1 year was the highest in Beqaa (87.5%, OR = 7.23, 95% CI = 1.51–34.57 compared to Beirut), followed by the South (72.5%, OR = 2.72, 95% CI = 1.16–6.42 compared to Beirut). Continuation rates were also significantly higher in the years 2021 (72.4%, OR = 7.88, 95% CI = 1.31–47.43), 2022 (73.4%, OR = 9.5, 95% CI = 1.69–53.42), and 2023 (73.4%, OR = 8.3, 95% CI = 1.52–45.12), compared to 2018 (*p* = 0.001).

However, no predictors were associated with exclusive BF, and only the year of birth was significantly associated with continuation to 2 years of BF. The highest continuation rates were observed in 2020 (27.6%) and 2021 (32.0%) (*p* = 0.001).

### 4.4 Association Between Support Services and Education Procedures to BF and BF Practices

Table 3 shows the association between some HCP help procedures and the five outcomes of BF. Around a quarter of participants (*n* = 69) knew about the MoPH's national BF hotline, of whom only 26 used it. None of the BF practices were associated with the knowledge or use of the MoPH's hotline. However, receiving prenatal education about BF before pregnancy (OR = 1.81 [1.03–3.15]) and during the third trimester (OR = 1.71 [1.03–2.81]) were associated with higher odds of early initiation of BF. Also, SSC was higher among participants who received prenatal education during the third trimester (OR = 1.73 [1.07–2.79]). The main sources of prenatal education were gynecologists, lactation specialists, and midwives, who had significant positive influences on some BF practices. Specifically, seeking prenatal education from gynecologists and midwives increased SSC (OR = 2.42 [1.11–5.25] and OR = 2.45 [1.34–4.47], respectively). Additionally, prenatal education sought from lactation specialists was significantly associated with higher early initiation rates (OR = 1.76 [1.07–2.90]), yet lower exclusive BF rates (OR = 0.39 [0.24–0.64]).

**TABLE 3:** Bivariate analysis between some support services and education procedures and the five outcomes of breastfeeding (*n* = 280).

			Skin‐to‐skin					Early initiation					Exclusive breastfeeding at 6 months				
		Total	No *N* (%)	Yes *N* (%)	*p*	OR	95% confidence interval (CI)	No *N* (%)	Yes *N* (%)	p	OR	95% confidence interval (CI)	No *N* (%)	Yes *N* (%)	*p*	OR	95% confidence interval (CI)
Knowledge about the Ministry of Public Health's national breastfeeding hotline																	
No		211 (75.4)	96 (46.6)	110 (53.4)	0.974	1.01	0.58–1.74	76 (36.2)	134 (63.8)	0.308	1.36	0.75–2.46	95 (45.0)	116 (55.0)	0.302	0.75	0.44–1.29
Yes		69 (24.6)	32 (46.4)	37 (53.6)				20 (29.4)	48 (70.6)				36 (52.2)	33 (47.8)			
Ever using the Ministry of Public Health's national breastfeeding hotline																	
No		254 (90.7)	112 (44.8)	118 (55.2)	0.072	0.46	0.19–1.07	85 (33.7)	167 (66.3)	0.383	0.69	0.31–1.58	117 (46.1)	137 (53.9)	0.45	0.73	0.33–1.64
Yes		26 (9.3)	16 (64.0)	9 (36.0)				11 (42.3)	15 (57.7)				14 (53.9)	33 (46.1)			
Time of receiving prenatal education about breastfeeding																	
Before pregnancy	No	191 (68.2)	95 (50.3)	94 (49.7)	0.068	1.62	0.97–2.73	73 (38.6)	116 (61.4)	0.038*	1.81	1.03–3.15	83 (43.5)	108 (56.5)	0.103	0.66	0.40–1.09
	Yes	89 (31.8)	33 (38.4)	53 (61.6)				23 (25.8)	66 (74.2)				48 (53.9)	41 (46.1)			
First trimester	No	203 (72.5)	94 (46.8)	107 (53.2)	0.904	1.03	0.61–1.76	74 (36.8)	127 (63.2)	0.197	1.46	0.82–2.58	90 (44.3)	113 (55.7)	0.183	0.7	0.41–1.18
	Yes	77 (27.5)	34 (46.0)	40 (54.0)				22 (28.6)	55 (71.4)				41 (53.2)	36 (46.8)			
Second trimester	No	158 (56.4)	76 (49.0)	79 (51.0)	0.348	1.26	0.78–2.03	57 (36.1)	101 (63.9)	0.535	1.17	0.71–1.94	66 (41.8)	92 (58.2)	0.056	0.62	0.39–1.01
	Yes	122 (43.6)	52 (43.3)	68 (56.7)				39 (32.5)	81 (67.5)				65 (53.3)	57 (46.7)			
Third trimester	No	142 (50.7)	74 (53.2)	65 (46.8)	0.025*	1.73	1.07–2.79	57 (40.4)	84 (59.6)	0.037*	1.71	1.03–2.81	69 (48.6)	73 (51.4)	0.539	1.16	0.72–1.85
	Yes	138 (49.3)	54 (39.7)	82 (60.3)				39 (28.5)	98 (71.5)				62 (44.9)	76 (55.1)			
Place for receiving prenatal education about breastfeeding																	
Private clinic	No	256 (91.4)	121 (48.0)	131 (52.0)	0.112	2.11	0.84–5.31	91 (35.8)	163 (64.2)	0.148	2.12	0.77–5.87	121 (47.3)	135 (52.7)	0.6	1.25	0.54–2.93
	Yes	24 (8.6)	7 (30.4)	16 (69.6)				5 (20.8)	19 (79.2)				10 (41.7)	14 (58.3)			
Primary healthcare center	No	271 (96.8)	125 (47.0)	141 (53.0)	0.51	—	—	93 (34.6)	176 (65.4)	1	—	—	124 (45.8)	147 (54.2)	0.088	—	—
	Yes	9 (3.2)	3 (33.3)	6 (66.7)				3 (33.3)	6 (66.7)				7 (77.8)	2 (22.2)			
Hospital	No	224 (80.0)	110 (50.0)	110 (50.0)	0.023*	2.06	1.10–3.83	81 (36.5)	141 (63.5)	0.175	1.57	0.82–3.01	110 (49.1)	114 (50.9)	0.121	1.61	0.88–2.93
	Yes	56 (20.0)	18 (32.8)	37 (67.3)				15 (26.8)	41 (73.2)				21 (37.5)	35 (62.5)			
Lactation consultant clinic	No	195 (69.6)	99 (52.1)	91 (47.9)	0.006*	2.10	1.24–3.57	77 (39.9)	116 (60.1)	0.005*	2.31	1.28–4.14	78 (40.0)	117 (60.0)	0.001*	0.4	0.24–0.68
	Yes	85 (30.4)	29 (34.1)	56 (65.9)				19 (22.4)	66 (77.7)				53 (62.3)	32 (37.7)			
Educational session in public places	No	226 (80.7)	103 (46.0)	121 (54.0)	0.695	0.89	0.48–1.63	78 (34.5)	148 (65.5)	0.989	0.99	0.53–1.88	104 (46.0)	122 (54.0)	0.598	0.85	0.47–1.54
	Yes	54 (19.3)	25 (49.0)	26 (51.0)				18 (34.6)	34 (65.4)				27 (50.0)	27 (50.0)			
Mom to mom support group	No	165 (58.9)	69 (42.3)	94 (57.7)	0.092	0.66	0.41–1.07	52 (31.7)	112 (68.3)	0.235	0.74	0.45–1.22	70 (42.4)	95 (57.6)	0.08	0.65	0.40–1.05
	Yes	115 (41.1)	59 (52.7)	53 (47.3)				44 (38.6)	70 (61.4)				61 (53.0)	54 (47.0)			
Personnel for receiving prenatal education about breastfeeding																	
Lactation consultant	No	128 (45.7)	64 (51.2)	61 (48.8)	0.158	1.41	0.87–2.27	53 (41.4)	75 (58.6)	0.027*	1.76	1.07–2.90	44 (34.4)	84 (65.6)	< 0.001*	0.39	0.24–0.64
	Yes	152 (54.3)	64 (42.7)	86 (57.3)				43 (28.7)	107 (71.3)				87 (57.2)	65 (42.8)			
Pediatrician	No	251 (89.6)	117 (47.4)	130 (52.6)	0.418	1.39	0.63–3.09	85 (34.1)	164 (65.9)	0.684	0.85	0.38–1.88	118 (47.0)	133 (53.0)	0.823	1.09	0.50–2.36
	Yes	29 (10.4)	11 (39.3)	17 (60.7)				11 (37.9)	18 (62.1)				13 (44.8)	16 (55.2)			
Gynecologist	No	243 (86.8)	118 (49.2)	122 (50.8)	0.026*	2.42	1.11–5.25	88 (36.2)	155 (63.8)	0.125	1.92	0.83–4.40	114 (46.9)	129 (53.1)	0.912	1.04	0.52–2.08
	Yes	37 (13.2)	10 (28.6)	25 (71.4)				8 (22.9)	27 (77.1)				17 (45.9)	20 (54.1)			
Midwife	No	215 (76.8)	109 (51.4)	103 (48.6)	0.004*	2.45	1.34–4.47	80 (37.6)	133 (62.4)	0.057	1.84	0.98–3.45	100 (46.5)	115 (53.5)	0.867	0.95	0.55–1.66
	Yes	65 (23.2)	19 (30.2)	44 (69.8)				16 (24.6)	49 (75.4)				31 (47.7)	34 (52.3)			
Nurse	No	235 (83.9)	112 (48.7)	118 (51.3)	0.109	1.72	0.89–3.33	81 (34.8)	152 (65.2)	0.853	1.07	0.54–2.09	115 (48.9)	120 (51.1)	0.102	1.74	0.90–3.37
	Yes	45 (16.1)	16 (35.6)	29 (64.4)				15 (33.3)	30 (66.7)				16 (35.6)	29 (64.4)			
Dietician	No	259 (92.5)	118 (46.5)	136 (53.5)	0.918	0.95	0.39–2.33	89 (34.6)	168 (65.4)	0.904	1.06	0.41–2.72	122 (47.1)	137 (52.9)	0.708	1.19	0.48–2.91
	Yes	21 (7.5)	10 (47.6)	11 (52.4)				7 (33.3)	14 (66.7)				9 (42.9)	12 (57.1)			
Experienced mom	No	138 (49.3)	53 (39.0)	83 (61.0)	0.013*	0.54	0.34–0.88	40 (29.2)	97 (70.8)	0.066	0.63	0.38–1.03	67 (48.6)	71 (51.5)	0.56	1.15	0.72–1.84
	Yes	142 (50.7)	75 (54.0)	64 (46.0)				56 (39.7)	85 (60.3)				64 (45.1)	78 (54.9)			
Personnel for receiving breastfeeding social support																	
Family members	No	56 (20.0)	27 (48.2)	29 (51.8)	0.779	1.09	0.60–1.96	24 (42.9)	32 (57.1)	0.144	1.56	0.86–2.84	25 (44.6)	31 (55.4)	0.719	0.90	0.50–1.62
	Yes	224 (80.0)	101 (46.1)	118 (53.9)				72 (32.4)	150 (67.5)				106 (47.3)	118 (52.7)			
Partner	No	45 (16.1)	21 (47.7)	23 (52.3)	0.864	1.06	0.55–2.02	16 (36.4)	28 (63.6)	0.781	1.10	0.56–2.15	20 (44.4)	25 (55.6)	0.731	0.89	0.47–1.70
	Yes	235 (83.9)	107 (46.3)	124 (53.7)				80 (34.2)	154 (65.8)				111 (47.2)	124 (52.7)			
Friends	No	88 (31.4)	41 (47.1)	46 (52.9)	0.895	1.03	0.62–1.72	32 (36.4)	56 (63.6)	0.662	1.13	0.66–1.91	43 (48.9)	45 (51.1)	0.637	1.13	0.68–1.87
	Yes	192 (68.6)	87 (46.3)	101 (53.7)				64 (33.7)	126 (66.3)				88 (45.8)	104 (54.2)			
Healthcare professional	No	117 (41.8)	58 (50.0)	58 (50.0)	0.327	1.27	0.79–2.05	42 (35.9)	75 (64.1)	0.683	1.11	0.67–1.83	51 (43.6)	66 (56.4)	0.8	0.36	0.50–1.29
	Yes	163 (58.2)	70 (44.0)	89 (56.0)				54 (33.5)	107 (66.5)				80 (49.1)	83 (50.9)			
Maternity leave																	
Less than 1 month		8 (5.6)	3 (5.1)	5 (6.0)	0.479			1 (2.1)	7 (7.4)	0.716			2 (2.9)	6 (8.0)	0.390		
1 month		12 (8.4)	7 (11.9)	5 (6.0)				4 (8.5)	8 (8.4)				4 (5.9)	8 (10.7)			
70 days		87 (25.2)	37 (62.7)	50 (59.5)				29 (61.7)	57 (60.0)				44 (64.7)	43 (57.3)			
More than 70 days		36 (25.2)	12 (20.3)	24 (28.5)				13 (27.7)	23 (24.2)				18 (26.5)	18 (24.0)			
Support in workplace																	
No		26 (22.2)	12 (23.5)	14 (21.2)	0.765			14 (34.1)	12 (16.0)	0.025*			6 (9.8)	20 (35.7)	0.001*		
Yes		91 (77.8)	39 (76.5)	52 (78.8)				27 (65.9)	63 (84.0)				55 (90.2)	36 (64.3)			

			Breastfeeding continuation to 1 year					Breastfeeding continuation to 2 years				
		Total	No *N* (%)	Yes *N* (%)	*p*	OR	95% confidence interval (CI)	No *N* (%)	Yes *N* (%)	*p*	OR	95% confidence interval (CI)
Knowledge about the Ministry of Public Health's national breastfeeding hotline												
No		211 (75.4)	95 (50.8)	92 (49.2)	< 0.001*	3.24	1.69–6.19	163 (87.2)	24 (12.8)	0.122	1.80	0.85–3.80
Yes		69 (24.6)	15 (24.2)	47 (75.8)				49 (79.0)	13 (21.0)			
Ever using the Ministry of Public Health's national breastfeeding hotline												
No		254 (90.7)	103 (46.0)	121 (54.0)	0.092	2.19	0.88–5.45	194 (86.6)	30 (13.4)	0.071		
Yes		26 (9.3)	7 (28.0)	18 (72.0)				18 (72.0)	7 (28.0)			
Time of receiving prenatal education about breastfeeding												
Before pregnancy	No	191 (68.2)	77 (45.6)	92 (54.4)	0.522	1.19	0.70–2.04	146 (86.4)	23 (13.6)	0.421	1.35	0.65–2.78
	Yes	89 (31.8)	33 (41.3)	47 (58.7)				66 (82.5)	14 (17.5)			
First trimester	No	203 (72.5)	83 (46.4)	96 (53.6)	0.265	1.38	0.78–2.42	157 (87.7)	22 (12.3)	0.072	1.95	0.94–4.02
	Yes	77 (27.5)	27 (38.6)	43 (61.4)				55 (78.6)	15 (21.4)			
Second trimester	No	158 (56.4)	64 (45.1)	78 (54.9)	0.744	1.09	0.66–1.80	123 (86.6)	19 (13.4)	0.451	1.31	0.65–2.64
	Yes	122 (43.6)	46 (43.0)	61 (57.0)				89 (83.2)	18 (16.8)			
Third trimester	No	142 (50.7)	59 (46.8)	67 (53.2)	0.395	1.24	0.75–2.05	106 (84.1)	20 (15.9)	0.649	0.85	0.42–1.71
	Yes	138 (49.3)	51 (41.5)	72 (58.5)				106 (86.2)	17 (13.8)			
Place for receiving prenatal education about breastfeeding												
Private clinic	No	256 (91.4)	99 (43.0)	131 (57.0)	0.216	0.55	0.21–1.42	193 (83.9)	37 (16.1)	0.086	—	—
	Yes	24 (8.6)	11 (57.9)	8 (42.2)				19 (100.0)	0 (0.0)			
Primary healthcare center	No	271 (96.8)	106 (44.0)	135 (56.0)	0.735	—	—	204 (84.7)	37 (15.3)	0.609	—	—
	Yes	9 (3.2)	4 (50.0)	4 (50.0)				8 (100.0)	0 (0.0)			
Hospital	No	224 (80.0)	86 (43.4)	112 (56.6)	0.642	0.86	0.47–1.60	167 (84.3)	31 (15.7)	0.487	0.72	0.28–1.83
	Yes	56 (20.0)	24 (47.1)	27 (52.9)				45 (88.2)	6 (11.8)			
Lactation consultant clinic	No	195 (69.6)	78 (45.4)	94 (54.7)	0.578	1.17	0.68–2.01	150 (87.2)	22 (12.8)	0.173	1.65	0.80–3.39
	Yes	85 (30.4)	32 (41.6)	45 (58.4)				62 (80.5)	15 (19.5)			
Educational session in public places	No	226 (80.7)	88 (44.2)	111 (55.8)	0.978	1.01	0.54–1.88	166 (83.4)	33 (16.6)	0.136	0.44	0.15–1.30
	Yes	54 (19.3)	22 (44.0)	28 (56.0)				46 (92.0)	4 (8.0)			
Mom to mom support group	No	165 (58.9)	71 (48.6)	75 (51.4)	0.093	1.55	0.93–2.60	129 (88.4)	17 (11.6)	0.092	1.83	0.91–3.69
	Yes	115 (41.1)	39 (37.9)	64 (62.1)				83 (80.6)	20 (19.4)			
Personnel for receiving prenatal education about breastfeeding												
Lactation consultant	No	128 (45.7)	53 (46.9)	60 (53.1)	0.430	1.22	0.74–2.02	95 (84.1)	18 (15.9)	0.665	0.86	0.43–1.72
	Yes	152 (54.3)	57 (41.9)	79 (58.1)				117 (86.0)	19 (14.0)			
Pediatrician	No	251 (89.6)	97 (43.3)	127 (56.7)	0.408	0.71	0.31–1.61	191 (85.3)	33 (14.7)	0.773	—	—
	Yes	29 (10.4)	13 (52.0)	12 (48.0)				21 (84.0)	4 (16.0)			
Gynecologist	No	243 (86.8)	96 (44.4)	120 (55.6)	0.828	1.09	0.52–2.28	184 (85.2)	32 (14.8)	1.000	—	—
	Yes	37 (13.2)	14 (42.4)	19 (57.6)				28 (84.9)	5 (15.2)			
Midwife	No	215 (76.8)	80 (41.9)	111 (58.1)	0.188	0.67	0.37–1.21	159 (83.3)	32 (16.8)	0/135	0.47	0.17–1.26
	Yes	65 (23.2)	30 (51.7)	28 (48.3)				53 (91.4)	5 (8.6)			
Nurse	No	235 (83.9)	90 (42.7)	121 (57.4)	0.256	0.67	0.33–1.34	177 (83.9)	34 (16.1)	0.200	0.45	0.13–1.53
	Yes	45 (16.1)	20 (52.6)	18 (47.4)				35 (92.1)	3 (7.9)			
Dietician	No	259 (92.5)	101 (44.1)	128 (55.9)	0.938	0.96	0.38–2.42	195 (85.2)	34 (14.8)	1.000	—	
	Yes	21 (7.5)	9 (45.0)	11 (55.0)				17 (85.0)	3 (15.0)			
Experienced mom	No	138 (49.3)	52 (43.3)	68 (56.7)	0.796	0.94	0.57–1.54	105 (87.5)	15 (12.5)	0.314	1.44	0.71–2.93
	Yes	142 (50.7)	58 (45.0)	71 (55.0)				107 (82.9)	22 (17.1)			
Personnel for receiving breastfeeding social support												
Family members	No	56 (20.0)	22 (46.8)	25 (53.2)	0.687	1.14	0.60–2.16	38 (80.9)	9 (15.1)	0.361	0.68	0.30–1.56
	Yes	224 (80.0)	88 (43.6)	114 (56.4)				174 (86.1)	28 (13.9)			
Partner	No	45 (16.1)	14 (35.9)	25 (64.1)	0.259	0.67	0.33–1.35	31 (79.5)	8 (20.5)	0.283	0.62	0.26–1.48
	Yes	235 (83.9)	96 (45.7)	114 (54.3)				181 (86.2)	29 (13.8)			
Friends	No	88 (31.4)	37 (45.7)	44 (54.3)	0.740	1.09	0.64–1.86	67 (82.7)	14 (17.3)	0.456	0.76	0.37–1.57
	Yes	192 (68.6)	73 (43.5)	95 (56.6)				145 (86.3)	23 (13.7)			
Healthcare professional	No	117 (41.8)	49 (47.1)	55 (52.9)	0.429	1.23	0.74–2.04	87 (83.7)	17 (16.4)	0.577	0.82	0.41–1.65
	Yes	163 (58.2)	61 (42.1)	84 (57.9)				125 (86.2)	20 (13.8)			
Maternity Leave												
Less than 1 month		5 (3.8)	0 (0.0)	5 (7.4)	0.062			4 (3.4)	1 (7.1)	0.568		
1 month		11 (8.4)	3 (4.8)	8 (11.8)				10 (8.6)	1 (7.1)			
70 days		81 (61.8)	42 (66.7)	39 (57.4)				71 (60.7)	10 (71.5)			
More than 70 days		34 (26.0)	18 (28.5)	16 (23.5)				32 (27.3)	2 (14.3)			
Support in workplace												
No		24 (21.8)	16 (33.3)	8 (12.9)	0.010*			20 (20.4)	4 (33.3)	0.291		
Yes		86 (78.2)	32 (66.7)	54 (87.1)				78 (79.6)	8 (66.7)			

*Note:* *p < 0.05 indicating statistical significance.

### 4.5 Association Between Co‐Variates and BF Practices

Table 4 shows the multivariate logistic regression models for the five outcomes of BF. All multivariate models were significant with varying, yet reasonable goodness of fits: SSC (Pearson chi^2^ (176) = 185.35; *p* = 0.299), early initiation of BF (Pearson chi^2^ (99) = 89.66; *p* = 0.738), exclusive BF (Pearson chi^2^ (8) = 9.40; *p* = 0.310), BF continuation to 1 year (Pearson chi^2^ (98) = 119.73; *p* = 0.067), and BF continuation to 2 years (Pearson chi^2^ (15) = 13.95; *p* = 0.529). Accounting for the effect of other covariates, mothers who experienced a normal delivery were more likely to have SSC than mothers who had cesarean section deliveries (AOR = 6.08 [3.30–11.21]), while receiving prenatal education about BF from experienced moms was negatively associated with SSC (AOR = 0.49 [0.27–0.90]). Only the mode of delivery remained significantly associated with early initiation of BF (AOR = 4.07 [2.24–7.41]). Surprisingly, receiving prenatal education about BF from lactation specialists (AOR = 0.41 [0.24–0.70]) and support from the workplace (AOR = 0.76 [0.58–0.99]) were negatively associated with exclusive BF. Moreover, only mothers who gave birth in year 2021 were more likely to continue BF to 1 year than those who delivered in 2018 (AOR = 7.23 [1.24–42.31]), and those who knew about the MoPH's national BF hotline continued to BF to 1 year more (AOR = 2.28 [1.08–4.84]). None of the covariates were significantly associated with BF continuation to 2 years in the final model.

**TABLE 4:** Multivariate analysis between sociodemographic characteristics, some support services, and education procedures and the five outcomes of breastfeeding (*n* = 280).

Skin‐to‐skin				
		*p*	Adjusted odds ratio (AOR)	95% confidence interval (CI)
Smoking other types				
Nonsmoker			Ref.	
Previous smoker		0.441	2.20	0.30–16.35
Current smoker		0.176	5.08	0.48–53.50
Alcohol consumption				
None			Ref.	
Previous		0.801	1.11	0.49–2.53
Current		0.306	1.66	0.63–4.35
Body mass index (BMI) before pregnancy		0.952	1.01	0.76–1.34
Body mass index (BMI) during last month of pregnancy		0.010*	0.60	0.41–0.88
Delivery place				
Public hospital in Lebanon			Ref.	
Private hospital in Lebanon		0.724	1.47	0.17–12.37
Homebirth		0.64	0.40	0.01–17.94
Hospital outside Lebanon		—	1.00	—
Mode of delivery				
Cesarean section with/without general anesthesia			Ref.	
Normal Vaginal delivery with/without local anesthesia		< 0.001*	6.08	3.30–11.21
Time of receiving prenatal education about breastfeeding				
Third trimester	No		Ref.	
	Yes	0.221	1.48	0.79–2.76
Place for receiving prenatal education about breastfeeding				
Hospital	No		Ref.	
	Yes	0.105	1.87	0.88–3.99
Lactation consultant clinic	No		Ref.	
	Yes	0.274	1.46	0.74–2.86
Personnel for receiving prenatal education about breastfeeding				
Gynecologist	No		Ref.	
	Yes	0.607	2.33	0.94–5.76
Midwife	No		Ref.	
	Yes	0.242	1.57	0.74–3.32
Experienced mom	No		Ref.	
	Yes	0.022*	0.49	0.27–0.90
**Early initiation of breastfeeding**				
Alcohol consumption				
None			Ref.	
Previous		0.773	1.12	0.51–2.48
Current		0.050	2.96	0.99–8.74
Mode of delivery				
Cesarean section with/without general anesthesia			Ref.	
Normal Vaginal delivery with/without local anesthesia		< 0.001*	4.07	2.24–7.41
Time of receiving prenatal education about breastfeeding				
Before pregnancy	No		Ref.	
	Yes	0.194	1.50	0.81–2.78
Third trimester	No		Ref.	
	Yes	0.207	1.44	0.82–2.55
Place for receiving prenatal education about breastfeeding				
Lactation consultant clinic	No		Ref.	
	Yes	0.352	1.43	0.67–3.03
Personnel for receiving prenatal education about breastfeeding				
Lactation consultant	No		Ref.	
	Yes	0.891	1.05	0.55–2.00
Support at workplace				
No			Ref.	
Yes		0.519	0.90	0.66–1.23
**Exclusive breastfeeding at 6‐months**				
Place for receiving prenatal education about breastfeeding				
Lactation consultant clinic	No		Ref.	
	Yes	0.224	1,36	0.83–2.22
Personnel for receiving prenatal education about breastfeeding				
Lactation consultant	No		Ref.	
	Yes	0.001*	0.41	0.24–0.70
Support at workplace				
No			Ref.	
Yes		0.043*	0.76	0.58–0.99
**Breastfeeding continuation to 1 year**				
Number of family members living in household				
≤ 3			Ref.	
4		0.798	1.14	0.41–3.16
5		0.714	0.84	0.34–2.11
≥ 6		0.616	0.80	0.34–1.89
Year of last birth				
2018			Ref.	
2019		0.156	4.06	0.59–28.08
2020		0.085	5.03	0.80–31.64
2021		0.028*	7.23	1.24–42.31
2022		0.057	5.42	0.95–30.79
2023		0.882	0.88	0.16–4.91
2024		0.258	5.54	0.29–107.05
Taking dietary supplements				
Multivitamins	No		Ref.	
	Yes	0.860	0.94	0.49–1.82
Delivery place				
Public hospital in Lebanon			Ref.	
Private hospital in Lebanon		0.204	4.54	0.44–46.92
Homebirth		—	1	—
Hospital outside Lebanon		—	1	—
Knowledge about the Ministry of Public Health's national breastfeeding hotline				
No			Ref.	
Yes		0.032*	2.28	1.08–4.84
Support at workplace				
No			Ref.	
Yes		0.100	1.33	0.95–1.88
**Breastfeeding continuation to 2 years**				
Year of last birth				
2018			Ref.	
2019		0.440	2.57	0.23–28.33
2020		0.394	2.67	0.28–25.44
2021		0.259	3.54	0.39–31.67
2022		0.562	0.50	0.05–5.19
2023		0.406	0.36	0.03–4.04
2024		0.340	4.91	0.19–128.70
Taking dietary supplements				
Calcium	No		Ref.	
	Yes	0.398	0.55	0.13–2.23
Multivitamins	No		Ref.	
	Yes	0.474	0.68	0.24–1.93

*p < 0.05 indicating statistical significance.

## 5 Discussion

This study explored BF practices in a sample of Lebanese mothers, the general factors associated with BF practices, and the role of healthcare professional support in enhancing BF outcomes, including SSC, early initiation of BF, exclusive BF up to 6 months, and BF continuation up to one and 2 years postpartum. Through this investigation, the study aimed to identify the impact of maternal education and prenatal support on these key BF practices, offering insights into potential interventions to support BF rates in Lebanon. The study found significant associations between specific sociodemographic factors, healthcare professional support, and BF practices. SSC was notably more common among mothers with higher income levels, those who delivered in private hospitals, and those who received prenatal education from gynecologists, lactation specialists, and midwives. The mode of delivery was also significantly associated with SSC and early initiation of BF, with normal vaginal deliveries correlating with higher adherence to these practices. While SSC and early initiation of BF were positively linked to healthcare professional support, exclusive BF rates showed no significant association with sociodemographic or health factors. BF continuation rates were notably higher in the Beqaa and South governorates, and recent years showed an increase in BF continuation up to 2 years.

### 5.1 Sociodemographic and Economic Factors and BF Practices

In line with previous research, this study found that sociodemographic and economic characteristics were generally weak predictors of BF practices. While factors like maternal education, employment, or income may indirectly influence BF, they rarely serve as strong independent predictors (Kehinde et al. 2023). This observation aligns with findings from studies in the United States and Europe, where sociodemographic factors often have limited direct impact on BF exclusivity or duration despite various levels of BF education, emphasizing the role of healthcare support instead (Jesus et al. 2016).

However, income was a notable exception in this study, showing a significant association with SSC in the bivariate analysis, yet not in the multivariate model. Higher‐income and more educated mothers are more likely to experience immediate SSC after birth, as they often choose to deliver in private hospitals. These facilities generally provide more SSC opportunities and enhanced BF support compared with public hospitals. Additionally, private hospitals serving affluent and well‐educated populations are more likely to implement BF‐friendly policies, employ highly trained staff, and offer abundant resources that promote SSC and lactation. These results align with findings from a 2020 study in Lebanon, which highlighted that maternal awareness of baby‐friendly hospital practices is closely linked to higher income, advanced education, and previous positive SSC experiences (Oueidat et al. 2020). Similarly, a 2020 study in Brazil reported that mothers with higher socioeconomic status had better access to SSC and BF‐friendly practices due to their choice of private facilities, suggesting that private healthcare resources often provide more robust support than public hospitals (Kehinde et al. 2023).

A regional analysis from 2019 showed that total BF duration was significantly longer in Beirut, Beirut suburbs, North Lebanon, Bekaa, and South Lebanon compared with Mount Lebanon (5.35 vs. 4.62 months) (Mattar et al. 2019). This pattern aligns with this study's findings, which showed that BF continuation to 1 year was highest in Beqaa (87.5%) and South Lebanon (72.5%) (*p* = 0.007) compared to other regions like Beirut and Mount Lebanon. These differences may reflect potential cultural or religious influences that support and encourage extended BF practices in these regions. For instance, a 2012 study on BF practices in urban and rural Vietnam found that exclusive BF at 3 months of age was more prevalent in rural areas, highlighting the influence of traditional cultural norms in rural settings (Thu et al. 2012). Such cultural reinforcement of BF in Lebanon's rural regions may also be less affected by the aggressive formula marketing, which continues to be a substantial barrier in urban areas. A UNICEF report underscores this issue, noting that urban populations are more exposed to such marketing strategies, which highly influence infant feeding decisions (UNICEF and WHO 2022).

### 5.2 Birth Type and Year of Birth With BF Practices

This study also found that the mode of delivery played a significant role in BF practices, with normal vaginal deliveries, with or without anesthesia, being significantly associated with higher SSC and early BF initiation compared with cesarean sections. Similar findings were reported in Hungary, where a high rate of skin‐to‐skin contact was remarked with a significant difference between normal vaginal deliveries (91.2%), elective cesarean sections (38.7%), and emergency cesarean sections (27.3%) (*p* < 0.001) (Hulman et al. 2024). The delayed maternal–infant contact due to cesarean procedures limits opportunities for early bonding and colostrum feeding, which are critical for establishing BF practices (Xu et al. 2024). The WHO recommends that mother‐infant SSC should begin with direct contact with the mother's bare skin within 1 min of birth (WHO 2022). Recommendations from global health organizations have emphasized the need for policies that support SSC in cesarean deliveries by minimizing the mother‐baby separation time and training staff to provide BF support in operating and recovery rooms (Office of the Surgeon General 2011; Wolter Kluwer 2023). Increasing BF support in cesarean deliveries, as Lebanon's national initiatives intend to do as part of the BFHI implementation, could help mitigate these barriers.

BF continuation to 1 year was particularly high in recent years, with rates of 72.4% in 2021 and 73.4% in both 2022 and 2023. This increase may reflect changes brought on by the COVID‐19 pandemic, during which disruptions to health services, extended time at home, and reduced access to formula likely reinforced reliance on BF as a practical and accessible option for infant nutrition. In addition to the shifts prompted by the COVID‐19 pandemic, Lebanon has been enduring a prolonged economic crisis. In 2021, this crisis led to severe shortages in baby formula, driving families unable to secure it reliably to adopt BF as a more accessible, resilient, and sustainable alternative amidst persistent financial hardships (Tabbara 2021). Continuation rates up to 2 years also rose during this period, peaking at 27.6% in 2020 and 32.0% in 2021, possibly due to reduced access to in‐person lactation support and healthcare resources, leaving mothers with fewer alternatives and encouraging them to continue BF. A 2023 study indicated that during the pandemic, women were more likely to exclusively breastfeed for 6 months compared with periods before or after, underscoring the positive effect of circumstances, such as extended maternal and paternal leave, which allow parents more time for bonding with infants (Jones et al. 2023). Additionally, the intention to breastfeed emerged as the strongest predictor of 6‐month BF duration, suggesting that prenatal interventions promoting and encouraging BF could further sustain these increased rates (Jones et al. 2023).

### 5.3 Impact of Professional Support on BF Practices

HCPs' role in supporting BF was evident, as prenatal education provided by gynecologists, lactation specialists, and midwives was significantly associated with positive BF outcomes. This study sheds light on critical gaps and strengths in BF practices in Lebanon, particularly regarding the influence of HCPs on BF outcomes. One notable finding revealed that only 24.6% of participants were aware of the MoPH's national BF hotline, and among those aware, just 37.7% used it. Interestingly, the low outreach to the MoPH's BF national hotline may explain why this study found no significant association between BF practices and knowledge or usage of the MoPH's hotline. This finding contrasts with evidence from other regions, showing that effective hotlines, when fully integrated into healthcare support systems, positively influence BF outcomes (Appalachian Breastfeeding Network 2024; Tennessee Department of Health 2024). This low utilization likely stems from the limited visibility and promotion of the hotline, suggesting a need for more targeted outreach strategies to ensure that mothers recognize the hotline as a supportive resource during BF. According to the CDC (2013), BF support resources, including hotlines, need widespread and strategic promotion to be recognized as credible sources of assistance. Effective outreach could involve hospital‐based communication, digital campaigns, and leveraging community channels. Additionally, integrating information about the hotline into prenatal educational materials could ensure that mothers become familiar with this resource during their pregnancy journey, thereby encouraging its use and BF adherence.

More importantly, one‐third of the sampled population accessed lactation support from a BF specialist or consultant. Support from lactation specialists increased early BF initiation, indicating the effective promotion of early BF initiation. Moreover, this study revealed that prenatal education provided by gynecologists, lactation specialists, and midwives, particularly during the third trimester, was significantly associated with higher rates of early BF initiation and SSC. Specifically, mothers who received prenatal BF education during the third trimester had increased odds of early BF initiation and SSC, emphasizing the possible role of late‐pregnancy BF education in preparing mothers for successful BF practices. This finding aligns with the CDC's recommendations on the importance of late‐pregnancy education to maximize BF success.

These findings highlight the critical need to strengthen and expand BF initiatives across Lebanon, mainly by empowering HCPs with enhanced training and resources. HCPs play a vital role in supporting practices, such as SSC and exclusive BF, particularly through programs like the BFHI. The BFHI encourages HCPs to adopt standardized BF practices, improving consistency in BF support across public and private hospitals and helping to close socioeconomic gaps (Office of the Surgeon General 2011). HCPs, including doctors, nurses, and midwives who work in maternity, neonatal, and pediatric settings, must be equipped with skills in lactation counseling and early bonding techniques to address barriers such as early formula supplementation and limited postnatal support (Wolter Kluwer 2023). Training initiatives that emphasize SSC immediately after birth and rooming‐in can significantly improve BF initiation rates, as recommended by the WHO (Kehinde et al. 2023). In Lebanon, many health workers lack adequate education in lactation care, leading to inconsistent or outdated guidance for new mothers. By integrating the Baby‐Friendly Hospital Initiative's (BFHI) 40‐h training modules and regular refresher courses into hospital staff development programs, HCPs can gain crucial hands‐on skills to assist mothers with BF techniques, such as positioning and resolving latch issues. Additionally, BF education should be included in the curricula of health‐related schools and incorporated into ongoing professional development programs (Akik et al. 2015). HCPs can also extend their impact through community partnerships that enable ongoing BF support. For example, establishing a network with local organizations and NGOs to offer mother‐to‐mother support and peer counseling can help mothers continue BF after leaving the hospital, addressing one of the most critical periods for BF continuation (Office of the Surgeon General 2011). Collaborations with community‐based lactation support groups and a well‐publicized national BF hotline could provide the accessible, culturally sensitive support mothers need postpartum. Studies highlight that such structured community support effectively bridges the gap between hospital discharge and long‐term BF success (Kehinde et al. 2023).

### 5.4 Structural Barriers to Exclusive BF

This study highlighted a percentage of exclusive BF (53.2%) that exceeds previously published national rates and international literature, showing that while BF initiation rates in Lebanon are high, fewer than 20% of mothers continue exclusive BF for the full 6‐month period (Chehab et al. 2020). This is probably due to sample‐specific factors, such as high educational level and socioeconomic status; nevertheless, these factors did not show a statistical association with exclusive BF, underscoring no gap between the rich and poor or educated and illiterate addressed in other studies (Mawa et al. 2019; Mututho et al. 2017). This highlights the need for further investigation to examine mediating and interacting effects between predictors and exclusive BF.

It has also shown limited predictions to exclusive BF from birth factors as well as some support services and education procedures. While prenatal education in Lebanon is vital for encouraging mothers to initiate BF, its effectiveness in helping mothers sustain exclusive BF for the recommended 6 months is limited and unexpected. This limitation is primarily due to various external factors, such as prevailing social and cultural norms, financial pressures that necessitate an early return to work, and a lack of adequate postnatal support. Also, family members and friends often send mixed messages. In addition, it indicates the necessity of emphasizing the WHO recommendation of exclusive BF by 6 months by lactation specialists and, more importantly, pediatricians who might encourage mothers to start solid food by 4 months.

Moreover, support from lactation specialists was correlated with lower exclusive BF rates by 6 months. This observation suggests that lactation specialists fell short in addressing the maintenance of exclusive BF for 6 months, and this may require additional support. This outcome may reflect the unique challenges faced by first‐time mothers or primigravida, who may initiate BF but struggle to maintain exclusivity without sustained postnatal support (Devi et al. 2022). Indeed, first‐time mothers often face unique challenges in sustaining exclusive BF, such as latching difficulties and concerns about milk supply. Findings from a 2023 study support this notion, revealing that 20% of primigravida mothers ceased BF within the first 6 months, primarily due to perceived insufficient milk supply and concerns over infant weight gain (Oberfichtner et al. 2023). Therefore, continuous postnatal support is crucial for long‐term BF success, especially among younger mothers or those returning to work early, as they find more difficulties maintaining exclusive BF (Jesus et al. 2016). Hence, implementing follow‐up sessions with lactation specialists and providing accessible resources addressing common BF challenges could help in sustaining BF exclusivity.

### 5.5 Strengths and Limitations

This study covered participants from all over Lebanon, used validated tools in the Lebanese context, addressed different BF practices, and shed light on a national priority. Moreover, it focused on the essentiality of integrating HCPs in lactation and BF‐related policies, which are usually overlooked in research and practice. Nevertheless, some limitations associated with the study should be acknowledged. The online survey may introduce selection bias by missing insights from non‐Internet users. However, it still captured a diverse sample of mothers with varying educational backgrounds, employment statuses, and socioeconomic experiences. Future studies should include more diverse samples recruited from households or healthcare facilities. Selection bias may affect the external validity of the study, meaning the sample may not be representative. However, generalizability is not the main focus of this research, which primarily aims to evaluate national strategic initiatives for breastfeeding (BF) and assess certain tools. Since responses were self‐reported, participants were not observed while completing the questionnaire, and some characteristics, such as height, weight, and weeks of pregnancy, might lack accuracy. Surveys are also subject to potential recall bias. These factors are expected to cause a nondifferential information bias, driving the results toward the null and underestimating some associations. Moreover, the cross‐sectional design of the study prevents establishing causal relationships between variables such as prenatal education and BF practices. Also, we acknowledge that this study did not explore the impact of structured training programs for HCPs due to the scope of the overarching study that targets mothers; yet this should be further investigated in future studies. Furthermore, despite conducting multivariable analysis, residual confounding due to unmeasured potential confounders might still be possible. Further studies that account for all these weaknesses are warranted to confirm this study's findings.

### 5.6 Public Health Implications

The multisectoral nature of BF (involving health, labor, and education sectors) means coordination is required in Lebanon. This study's findings underscore a need for structured BF support covering both pre‐ and postnatal stages, with HCPs like gynecologists, midwives, nurses, and lactation consultants playing key roles in this continuum of care. Systematic reviews have shown that training HCPs to provide consistent BF support postdischarge can increase BF duration and exclusivity, emphasizing the value of continuity of care (Blixt et al. 2023). Predelivery education equips mothers with essential knowledge about BF benefits, latch techniques, and milk production, laying a strong foundation (Kehinde et al. 2023; Obstetricians and Gynecologists 2016). Postdelivery support provides ongoing reassurance and assistance in addressing challenges, which is particularly impactful during the first few weeks postpartum when BF habits are established (Obstetricians and Gynecologists 2016).

While the MoPH has shown commitment (e.g., by initiating the national IYCF program, BFHI, and taking legal action against some violators of law 47/2008), the follow‐through has been inconsistent. From a policy perspective, the priority should be to consolidate these measures into a unified national framework, supported by adequate resources and strong accountability mechanisms. This involves rigorously implementing existing legislation, such as Law 47/2008, to restrict inappropriate formula marketing. Additionally, labor policies should be revised to better accommodate BF mothers. Engaging key stakeholders and including healthcare providers, professional associations, NGOs, international NGOs, and community leaders in a collaborative effort is crucial for enhancing maternal and child nutrition.

Finally, the status of a hospital as baby‐friendly directly affects the quality and extent of BF support. Hospitals designated as “Baby‐Friendly” follow the WHO and UNICEF's BFHI guidelines, which ensure staff are adequately trained in BF practices, promote SSC immediately after birth, and encourage exclusive BF from the start (CDC 2013). Conversely, nonbaby‐friendly hospitals may lack the same level of structured support and training, which can affect staff's ability to offer consistent BF guidance, potentially leading to lower initiation and continuation rates. In Lebanon, increasing the number of BFHI‐certified hospitals and training staff in accordance with BFHI standards could provide mothers with a more supportive BF environment, further promoting exclusive BF practices.

## 6 Conclusion

This paper introduces the methodology for a series of studies using online‐based survey data and presents updated rates of BF practices among mothers with children under 5 years of age. The findings reveal an increase in BF rates following the implementation of several national policies; however, these rates remain suboptimal. Notably, lactation support from different healthcare professionals (HCPs), that is, gynecologists and lactation consultants, was significantly associated with BF practices. These findings indicate the need for more emphasis on implementing national BF policies, raising awareness about the MoPH's hotline, and establishing structured BF support in both prenatal and postnatal stages, with active involvement from HCPs. Moreover, integrating standardized BF training, such as the WHO/UNICEF 40‐h BFHI course, into the accreditation criteria for hospitals can enhance consistent lactation support across Lebanon's healthcare system. MoPH, in collaboration with professional organizations, could require this training for maternity, neonatal, and pediatric staff as part of the BFHI and hospital accreditation process. Although Lebanon's economic crisis presents challenges, affordable solutions such as online training, partnerships with NGOs, international NGOs, and donor support can help to overcome financial obstacles. Finally, framing BF education as a cost‐effective public health investment strengthens the argument for implementing these initiatives, where a unified national approach that aligns regulation, education, and resource mobilization would ensure that this initiative is both practical and sustainable.

## Author Contributions

**Bahia Abdallah:** investigation (lead), methodology (lead), project administration (lead), supervision (lead), writing – original draft (supporting). **Eman Sharara:** investigation (equal), software (supporting), visualization (lead), writing – original draft (supporting). **Petra Nicolas:** writing – review and editing (equal). **Hala Sacre:** writing – review and editing (equal). **Cosette Fakih El Khoury:** methodology (equal), writing – review and editing (equal). **Pascale Salameh:** writing – review and editing (equal). **Chadia Haddad:** writing – review and editing (equal). **Joanne Karam:** writing – review and editing (equal). **Rana Rizk:** investigation (supporting), methodology (supporting), writing – review and editing (equal).

## Conflicts of Interest

The authors declare no conflicts of interest.

## Supporting information


**Supporting Information**:

## Data Availability

The data that support the findings of this study are available from the corresponding author upon reasonable request.
