# Supplementary material for: Factors Associated With Breastfeeding Outcomes in Lebanon: A National Cross‐Sectional Study
Source: Food Sci Nutr. 2025 Aug 6;13(8):e70767. doi: 10.1002/fsn3.70767 (PMC12328244; doi:10.1002/fsn3.70767)
Supplement: Supplementary file 1 — Supporting Information: [file FSN3-13-e70767-s001.zip › fsn370767-sup-0001-Supinfo1@Supplementary Material 1.docx]

*Supplementary Material 1: Bivariate analysis between the sociodemographic, economic, and health characteristics and the five outcomes of breastfeeding*

|  |  | **Skin-to-skin** | | | | | **Early initiation** | | | | | **Exclusive Breastfeeding at 6 months** | | | | |
| --- | --- | --- | --- | --- | --- | --- | --- | --- | --- | --- | --- | --- | --- | --- | --- | --- |
|  |  | *No*  *Mean ± S.D. or N (%)* | *Yes*  *Mean ± S.D. or N (%)* | *p-value* | *OR* | *95% Confidence interval (CI)* | *No*  *Mean ± S.D. or N (%)* | *Yes*  *Mean ± S.D. or N (%)* | *p-value* | *OR* | *95% Confidence interval (CI)* | *No*  *Mean ± S.D. or N (%)* | *Yes*  *Mean ± S.D. or N (%)* | *p-value* | *OR* | *95% Confidence interval (CI)* |
| **Age** |  | 31.5 ± 4.6 | 32.1 ± 4.7 | 0.343 | 1.02 | 0.97 – 1.08 | 31.7 ± 4.9 | 31.9 ± 4.6 | 0.774 | 1.01 | 0.96 - 1.06 | 32.2 ± 4.7 | 31.4 ± 4.7 | 0.178 | 0.97 | 0.92 - 1.02 |
| **Number of children** |  | 1.8 ± 0.8 | 1.6 ± 0.7 | 0.860 | 0.86 | 0.63 – 1.18 | 1.7 ± 0.8 | 1.6 ± 0.7 | 0.144 | 0.79 | 0.57 - 1.09 | 1.7 ± 0.7 | 1.6 ± 0.8 | 0.592 | 0.92 | 0.67 - 1.25 |
| **Number of children under 5 years** |  | 1.3 ± 0.5 | 1.3 ± 0.5 | 0.902 | 1.02 | 0.63 – 1.68 | 1.3 ± 0.5 | 1.3 ± 0.5 | 0.804 | 0.94 | 0.57 - 1.56 | 1.4 ± 0.5 | 1.3 ± 0.5 | 0.193 | 0.72 | 0.45 - 1.18 |
| **Educational level** |  |  |  | 0.302 |  |  |  |  | 0.346 |  |  |  |  | 0.146 |  |  |
| Intermediate or less |  | 4 (44.4) | 5 (55.6) |  | Ref. |  | 3 (30.0) | 7 (70.0) |  | Ref. |  | 5 (50.0) | 5 (50.0) |  | Ref. |  |
| Secondary and technical |  | 14 (48.3) | 15 (51.7) | 0.841 | 0.86 | 0.19 - 3.85 | 11 (37.9) | 18 (62.1) | 0.653 | 0.7 | 0.15 - 3.29 | 9 (31.0) | 20 (69.0) | 0.286 | 2.22 | 0.51 - 9.65 |
| University |  | 57 (52.3) | 52 (47.7) | 0.652 | 0.73 | 0.19 - 2.86 | 44 (39.3) | 68 (60.7) | 0.565 | 0.66 | 0.16 - 2.70 | 51 (45.5) | 61 (54.5) | 0.786 | 1.19 | 0.33 - 4.36 |
| Post-graduate |  | 53 (41.4) | 75 (58.6) | 0.858 | 1.13 | 0.29 - 4.42 | 38 (34.5) | 89 (65.5) | 0.996 | 1 | 0.25 - 4.09 | 66 (51.2) | 63 (48.8) | 0.944 | 0.95 | 0.26 - 3.46 |
| **Residence (by governorate)** |  |  |  | **0.020*** |  |  |  |  | 0.517 |  |  |  |  | 0.946 |  |  |
| Beirut |  | 26 (38.2) | 42 (61.8) |  | Ref. |  | 24 (35.3) | 44 (64.7) |  | Ref. |  | 31 (45.6) | 37 (54.4) |  | Ref. |  |
| Beqaa |  | 15 (60.0) | 10 (40.0) | 0.064 | 0.41 | 0.16 - 1.05 | 10 (40.0) | 15 (60.0) | 0.676 | 0.82 | 0.32 - 2.09 | 12 (48.0) | 13 (52.0) | 0.836 | 0.91 | 0.36 - 2.27 |
| Mount Lebanon |  | 48 (40.0) | 72 (60.0) | 0.812 | 0.93 | 0.50 - 1.71 | 35 (28.7) | 87 (71.3) | 0.346 | 1.36 | 0.72 - 2.55 | 58 (47.2) | 65 (52.9) | 0.835 | 0.94 | 0.52 - 1.70 |
| North Lebanon |  | 13 (61.9) | 8 (38.1) | 0.06 | 0.38 | 0.14 - 1.04 | 9 (40.9) | 13 (59.1) | 0.635 | 0.79 | 0.29 - 2.11 | 11 (50.0) | 11 (50.0) | 0.719 | 0.84 | 0.32 - 2.19 |
| South Lebanon |  | 26 (63.4) | 15 (36.6) | **0.012*** | 0.36 | 0.16 - 0.79 | 28 (34.5) | 23 (65.5) | 0.372 | 0.69 | 0.32 - 1.54 | 19 (45.2) | 23 (54.8) | 0.917 | 1.01 | 0.47 - 2.19 |
| **Number of family members living in household** |  |  |  | 0.155 |  |  |  |  | 0.152 |  |  |  |  | 0.459 |  |  |
| ≤3 |  | 51 (41.5) | 72 (58.5) |  | Ref. |  | 38 (30.2) | 88 (69.8) |  | Ref. |  | 53 (41.7) | 74 (58.3) |  | Ref. |  |
| 4 |  | 49 (50.0) | 49 (50.0) | 0.206 | 0.71 | 0.42 - 1.21 | 36 (36.7) | 62 (63.3) | 0.3 | 0.74 | 0.42 - 1.30 | 51 (52.0) | 47 (48.0) | 0.125 | 0.66 | 0.39 - 1.12 |
| 5 |  | 21 (56.8) | 16 (43.2) | 0.104 | 0.54 | 0.26 - 1.13 | 17 (44.7) | 21 (55.3) | 0.098 | 0.53 | 0.25 - 1.12 | 19 (50.0) | 19 (50.0) | 0.368 | 0.72 | 0.35 - 1.48 |
| ≥6 |  | 7 (46.7) | 8 (53.3) | 0.700 | 0.81 | 0.28 - 2.37 | 5 (35.7) | 9 (64.3) | 0.67 | 0.777 | 0.24 - 2.47 | 6 (40.0) | 9 (60.0) | 0.898 | 1.07 | 0.36 - 3.20 |
| **Number of rooms in the household** |  |  |  | 0.104 |  |  |  |  | 0.226 |  |  |  |  | 0.233 |  |  |
| ≤2 |  | 11 (57.9) | 8 (42.1) |  | Ref. |  | 9 (42.9) | 12 (57.1) |  | Ref. |  | 9 (42.9) | 12 (57.1) |  | Ref. |  |
| 3 |  | 32 (47.8) | 35 (52.2) | 0.437 | 1.5 | 0.54 - 4.21 | 25 (36.8) | 43 (63.2) | 0.616 | 1.29 | 0.48 - 3.49 | 37 (53.6) | 32 (46.4) | 0.389 | 0.65 | 0.24 - 1.74 |
| 4 |  | 45 (49.5) | 46 (50.6) | 0.504 | 1.41 | 0.52 - 3.82 | 31 (33.7) | 61 (66.3) | 0.43 | 1.48 | 0.56 - 3.88 | 45 (48.9) | 47 (51.1) | 0.617 | 0.78 | 0.30 - 2.04 |
| 5 |  | 30 (44.1) | 38 (55.9) | 0.291 | 1.74 | 0.62 - 4.87 | 24 (35.3) | 44 (64.7) | 0.531 | 1.38 | 0.51 - 3.73 | 28 (41.2) | 40 (58.8) | 0.891 | 1.07 | 0.39 - 2.88 |
| ≥6 |  | 10 (33.3) | 20 (66.7) | 0.094 | 2.75 | 0.84 - 8.99 | 7 (24.1) | 22 (75.9) | 0.166 | 2.36 | 0.70 - 7.93 | 12 (40.0) | 18 (60.0) | 0.838 | 1.13 | 0.36 - 3.49 |
| **Income** |  |  |  | **0.001*** |  |  |  |  | 0.199 |  |  |  |  | 0.378 |  |  |
| No income - <450$ |  | 41 (65.1) | 22 (34.9) |  | Ref. |  | 24 (36.9) | 41 (63.1) |  | Ref. |  | 28 (43.1) | 37 (56.9) |  | Ref. |  |
| 450$ - 999$ |  | 21 (44.7) | 26 (55.3) | **0.034*** | 2.31 | 1.06 - 5.00 | 20 (43.5) | 26 (56.5) | 0.487 | 0.76 | 0.35 - 1.64 | 24 (51.1) | 23 (48.9) | 0.403 | 0.73 | 0.34 - 1.54 |
| 1000$ - 2000$ |  | 25 (43.1) | 22 (56.9) | **0.016*** | 2.46 | 1.18 - 5.12 | 20 (33.3) | 40 (66.7) | 0.675 | 1.17 | 0.56 - 2.44 | 23 (37.7) | 38 (62.3) | 0.539 | 1.25 | 0.61 - 2.55 |
| >2000$ |  | 41 (38.3) | 55 (61.7) | **0.001*** | 3.00 | 1.57 - 5.74 | 32 (29.9) | 75 (70.1) | 0.342 | 1.37 | 0.71 - 2.63 | 56 (52.3) | 51 (47.7) | 0.24 | 0.69 | 0.37 - 1.28 |
| **Year of last birth** |  |  |  | 0.216 |  |  |  |  | 0.259 |  |  |  |  | 0.753 |  |  |
| 2018 |  | 3 (37.5) | 5 (62.5) |  | Ref. |  | 5 (55.6) | 4 (44.4) |  | Ref. |  | 2 (22.2) | 7 (77.8) |  | Ref. |  |
| 2019 |  | 8 (53.3) | 7 (46.7) | 0.472 | 0.53 | 0.09 - 3.03 | 6 (37.5) | 10 (62.5) | 0.386 | 2.08 | 0.39 - 10.95 | 8 (50.0) | 8 (50.0) | 0.185 | 0.29 | 0.04 - 1.82 |
| 2020 |  | 14 (46.7) | 16 (53.3) | 0.644 | 0.69 | 0.14 - 3.39 | 8 (27.6) | 21 (72.4) | 0.132 | 3.28 | 0.69 - 15.41 | 15 (50.0) | 15 (50.0) | 0.155 | 0.29 | 0.05 - 1.60 |
| 2021 |  | 30 (58.8) | 21 (41.2) | 0.268 | 0.42 | 0.09 - 1.95 | 22 (43.1) | 29 (56.9) | 0.493 | 1.64 | 0.39 - 6.86 | 28 (54.9) | 23 (45.1) | 0.088 | 0.23 | 0.04 - 1.24 |
| 2022 |  | 32 (47.1) | 36 (52.9) | 0.61 | 0.68 | 0.15 - 3.05 | 22 (32.4) | 46 (67.7) | 0.182 | 2.61 | 0.64 - 10.70 | 35 (50.7) | 34 (49.2) | 0.126 | 0.28 | 0.05 - 1.43 |
| 2023 |  | 39 (40.2) | 58 (59.8) | 0.881 | 0.89 | 0.20 - 3.95 | 31 (31.3) | 68 (68.7) | 0.152 | 2.74 | 0.69 - 10.92 | 39 (39.4) | 60 (60.6) | 0.321 | 0.44 | 0.09 - 2.23 |
| 2024 |  | 2 (33.3) | 4 (66.7) | 0.872 | 1.2 | 0.13 - 11.05 | 2 (33.3) | 4 (66.7) | 0.403 | 2.5 | 0.29 - 21.39 | 4 (66.7) | 2 (33.3) | 0.099 | 0.14 | 0.01 - 1.44 |
| **Occupation** |  |  |  | 0.515 |  |  |  |  | 0.28 |  |  |  |  | 0.603 |  |  |
| Housewife |  | 29 (50.9) | 28 (49.1) |  | Ref. |  | 25 (43.1) | 33 (56.9) |  | Ref. |  | 26 (44.8) | 32 (55.1) |  | Ref. |  |
| Employed/Freelancer/Contractor in a scientific or health field |  | 22 (44.0) | 28 (56.0) | 0.478 | 1.32 | 0.61 - 2.83 | 16 (30.8) | 36 (69.2) | 0.183 | 1.7 | 0 | 20 (38.5) | 32 (61.5) | 0.5 | 1.3 | 0.61 - 2.78 |
| Employed/Freelancer/Contractor in another field |  | 40 (45.5) | 48 (54.6) | 0.523 | 1.24 | 0.64 - 2.42 | 28 (31.8) | 60 (68.2) | 0.167 | 1.62 | 0.77 - 3.74 | 47 (52.2) | 43 (47.8) | 0.38 | 0.74 | 0.38 - 1.44 |
| Stopping work to take care of child |  | 31 (52.5) | 28 (47.5) | 0.858 | 0.94 | 0.45 - 1.94 | 24 (40.7) | 35 (59.3) | 0.79 | 1.1 | 0.82 - 3.22 | 28 (47.5) | 31 (52.5) | 0.775 | 0.89 | 0.43 - 1.86 |
| Self-employed in a scientific or health field |  | 5 (29.4) | 12 (70.6) | 0.126 | 2.49 | 0.78 - 7.97 | 2 (11.8) | 15 (88.2) | **0.029*** | 5.68 | 0.53 - 2.31 | 9 (52.9) | 8 (47.1) | 0.556 | 0.72 | 0.24 - 2.1 |
| Student/Trainee |  | 1 (25.0) | 3 (75.0) | 0.339 | 3.11 | 0.30 - 31.68 | 1 (25.0) | 3 (75.0) | 0.488 | 2.27 | 1.19 - 27.15 | 1 (25.1) | 3 (75.0) | 0.452 | 2.44 | 0.24 - 23.84 |
| **Cigarette smoking** |  |  |  | 0.274 |  |  |  |  | 0.863 |  |  |  |  | 0.909 |  |  |
| Non-smoker |  | 111 (48.9) | 116 (51.1) |  | Ref. |  | 82 (35.8) | 147 (64.2) |  | Ref. |  | 106 (45.9) | 125 (54.1) |  | Ref. |  |
| Previous smoker |  | 7 (29.7) | 17 (70.8) | 0.072 | 2.32 | 0.93 - 5.82 | 5 (20.0) | 20 (80.0) | 0.122 | 2.23 | 0.81 - 6.17 | 15 (60.0) | 10 (40.0) | 0.184 | 0.57 | 0.24 - 1.31 |
| Current smoker: ≤10 |  | 5 (38.5) | 8 (61.5) | 0.467 | 1.53 | 0.49 - 4.82 | 4 (30.8) | 9 (69.2) | 0.712 | 1.25 | 0.37 - 4.20 | 5 (38.5) | 8 (61.5) | 0.602 | 1.36 | 0.43 - 4.27 |
| Current smoker: ≥11 |  | 5 (45.5) | 6 (54.6) | 0.824 | 1.14 | 0.34 - 3.87 | 5 (45.5) | 6 (65.5) | 0.518 | 0.67 | 0.19 - 2.26 | 5 (45.5) | 6 (54.5) | 0.978 | 1.02 | 0.30 - 3.43 |
| **Waterpipe smoking** |  |  |  | 0.099 |  |  |  |  | 0.086 |  |  |  |  | 0.109 |  |  |
| Non-smoker |  | 73 (45.9) | 86 (54.1) |  | Ref. |  | 56 (34.8) | 105 (65.2) |  | Ref. |  | 80 (49.4) | 82 (50.6) |  | Ref. |  |
| Previous smoker |  | 15 (35.7) | 27 (64.3) | 0.238 | 1.53 | 0.76 - 3.09 | 9 (21.4) | 33 (78.6) | 0.103 | 1.96 | 0.87 - 4.38 | 20 (47.6) | 22 (52.4) | 0.839 | 1.07 | 0.54 - 2.12 |
| Current smoker: <1/week |  | 21 (60.0) | 14 (40.0) | 0.403 | 1.69 | 0.49 - 5.89 | 17 (48.6) | 18 (51.4) | 0.093 | 5.87 | 0.74 - 46.62 | 14 (38.9) | 22 (61.1) | 0.256 | 0.49 | 0.14 - 1.68 |
| Current smoker: 1-2/week |  | 4 (33.3) | 8 (66.7) | 0.583 | 0.74 | 0.26 - 2.15 | 1 (8.3) | 11 (91.7) | 0.477 | 0.69 | 0.24 - 1.94 | 8 (66.7) | 4 (33.3) | 0.368 | 1.62 | 0.56 - 4.68 |
| Current smoker: 3-6/week |  | 8 (53.3) | 7 (46.7) | 0.41 | 0.61 | 0.18 - 1.99 | 7 (43.8) | 9 (56.2) | 0.295 | 0.53 | 0.16 - 1.73 | 6 (37.5) | 10 (62.5) | 0.117 | 2.93 | 0.76 - 11.21 |
| Current smoker: ≥7/week |  | 7 (58.3) | 5 (41.7) | 0.134 | 0.57 | 0.27 - 1.19 | 6 (50.0) | 6 (50.0) | 0.129 | 0.56 | 0.27 - 1.18 | 3 (25.0) | 9 (75.0) | 0.256 | 1.53 | 0.73 -3.21 |
| **Alcohol consumption** |  |  |  | **0.005*** |  |  |  |  | **0.016*** |  |  |  |  | 0.675 |  |  |
| None |  | 105 (51.5) | 99 (48.5) | Ref. |  |  | 78 (38.1) | 127 (61.9) |  | Ref. |  | 95 (46.1) | 111 (53.9) |  | Ref. |  |
| Previous |  | 8 (28.6) | 20 (71.4) | 2.65 | 1.12 - 6.29 | **0.027*** | 5 (17.2) | 24 (82.8) | **0.035*** | 2.95 | 1.08 - 8.05 | 15 (50.0) | 15 (50.0) | 0.691 | 0.86 | 0.39 - 1.84 |
| Current |  | 15 (34.9) | 28 (65.1) | 1.98 | 0.99 - 3.93 | 0.051 | 13 (29.6) | 31 (70.5) | 0.29 | 1.46 | 0.72 - 2.97 | 21 (47.7) | 23 (52.3) | 0.846 | 0.94 | 0.49 - 1.79 |
| **Taking dietary supplements** |  |  |  |  |  |  |  |  |  |  |  |  |  |  |  |  |
| Iron | No | 94 (49.5) | 96 (50.5) |  | Ref. |  | 72 (37.5) | 120 (62.5) |  | Ref. |  | 90 (46.4) | 104 (53.6) |  | Ref. |  |
|  | Yes | 34 (40.0) | 51 (60.0) | 0.146 | 1.47 | 0.87 – 2.47 | 24 (27.9) | 62 (72.1) | 0.121 | 1.55 | 0.89 - 2.69 | 41 (47.7) | 45 (52.3) | 0.843 | 0.95 | 9.57 - 1.58 |
| Folic acid | No | 102 (46.8) | 116 (53.2) |  | Ref. |  | 75 (33.9) | 146 (66.1) |  | Ref. |  | 102 (45.7) | 121 (54.3) |  | Ref. |  |
|  | Yes | 26 (45.6) | 31 (54.4) | 0.874 | 1.05 | 0.58 – 1.88 | 21 (36.8) | 36 (63.2) | 0.681 | 0.88 | 0.48 - 1.61 | 29 (50.9.1) | 28 (49.1) | 0.488 | 0.81 | 0.46 - 1.46 |
| Vitamin D | No | 64 (45.7) | 76 (54.3) |  | Ref. |  | 52 (37.1) | 88 (62.9) |  | Ref. |  | 60 (42.3) | 82 (57.8) |  | Ref. |  |
|  | Yes | 64 (47.4) | 71 (52.6) | 0.778 | 0.93 | 0.58 – 1.50 | 44 (31.9) | 94 (68.2) | 0.357 | 1.26 | 0.77 - 2.07 | 71 (51.4) | 67 (48.6) | 0.124 | 0.69 | 0.43 - 1.11 |
| Omega 3 | No | 110 (46.8) | 125 (53.2) |  | Ref. |  | 81 (34.0) | 157 (66.0) |  | Ref. |  | 110 (45.8) | 130 (54.2) |  | Ref. |  |
|  | Yes | 18 (45.0) | 22 (55.0) | 0.832 | 1.08 | 0.55 – 2.11 | 15 (37.5) | 25 (62.5) | 0.67 | 0.86 | 0.43 - 1.72 | 21 (52.5) | 19 (47.5) | 0.435 | 0.77 | 0.39 - 1.49 |
| Calcium | No | 100 (47.2) | 112 (52.8) |  | Ref. |  | 74 (34.6) | 140 (65.4) |  | Ref. |  | 99 (45.8) | 117 (54.2) |  | Ref. |  |
|  | Yes | 28 (55.6) | 35 (23.8) | 0.703 | 1.12 | 0.63 – 1.96 | 22 (34.4) | 42 (65.6) | 0.976 | 1.01 | 0.56 - 1.82 | 32 (50.0) | 32 (50.0) | 0.558 | 0.85 | 0.48 - 1.48 |
| Multi-vitamins | No | 84 (47.7) | 92 (52.3) |  | Ref. |  | 58 (32.8) | 119 (67.2) |  | Ref. |  | 88 (49.2) | 91 (50.8) |  | Ref. |  |
|  | Yes | 44 (44.4) | 55 (55.6) | 0.600 | 1.14 | 0.69 – 1.87 | 38 (37.6) | 63 (62.4) | 0.413 | 0.81 | 0.49 - 1.35 | 43 (42.6) | 58 (57.4) | 0.289 | 1.3 | 0.79 - 2.13 |
| **Body Mass Index before pregnancy** |  | 24.0 ± 4.7 | 22.3 ± 3.8 | **0.001*** | 0.91 | 0.85 – 0.96 | 23.8 ± 4.8 | 22.8 ± 4.0 | 0.079 | 0.95 | 0.89 - 1.01 | 23.4 ± 4.7 | 22.4 ± 4.7 | 0.262 | 0.97 | 0.92 - 1.02 |
| **Body Mass Index during the last month of pregnancy** |  | 29.0 ± 5.1 | 27.2 ± 4.4 | **0.002*** | 0.92 | 0.87 – 0.97 | 28.7 ± 5.2 | 27.8 ± 4.6 | 0.162 | 0.96 | 0.92 - 1.01 | 28.3 ± 5.1 | 27.9 ± 4.5 | 0.421 | 0.98 | 0.93 - 1.03 |
| **Delivery place** |  |  |  | **0.031*** |  |  |  |  | 0.670 |  |  |  |  | 0.419 |  |  |
| Public hospital in Lebanon |  | 5 (100) | 0 (0.0) |  | Ref. |  | 1 (20.0) | 4 (80.0) |  | Ref. |  | 2 (40.0) | 3 (60.0) |  | Ref. |  |
| Private hospital in Lebanon |  | 121 (46.0) | 142 (54.0) | 0.276 | 0.29 | 0.03 – 2.66 | 93 (35.1) | 172 (64.9) | 0.493 | 0.46 | 0.05 - 4.19 | 126 (47.2) | 141 (52.8) | 0.75 | 0.75 | 0.12 - 4.54 |
| Homebirth |  | 1 (50.0) | 1 (50.0) | 0.442 | 0.25 | 0.01 – 8.56 | 1 (50.0) | 1 (50.0) | 0.442 | 0.25 | 0.01 - 8.56 | 2 (100.0) | 0 (0.0) | - | 1 | - |
| Hospital outside Lebanon |  | 1 (20.0) | 4 (80.0) | - | 1 | - | 1 (16.7) | 5 (83.3) | 0.887 | 1.25 | 0.06 - 26.87 | 1 (16.7) | 5 (83.3) | 0.398 | 3.33 | 0.20 - 54.53 |
| **Mode of delivery** |  |  |  |  |  |  |  |  |  |  |  |  |  |  |  |  |
| Cesarean section with/without general anesthesia |  | 101 (63.9) | 57 (36.1) | Ref. |  |  | 76 (47.2) | 85 (52.8) |  | Ref. |  | 79 (48.5) | 84 (51.5) |  | Ref. |  |
| Normal Vaginal delivery with/without local anesthesia |  | 27 (23.1) | 90 (76.9) | <0.001* | 5.91 | 3.45 – 10.12 | 20 (17.1) | 97 (82.9) | <0.001* | 4.34 | 2.45 - 7.68 | 52 (44.4) | 65 (55.6) | 0.506 | 1.18 | 0.73 - 1.89 |
| **Pregnancy period** |  |  |  |  |  |  |  |  |  |  |  |  |  |  |  |  |
| Pre-term |  | 38 (49.4) | 39 (50.6) |  | Ref. |  | 27 (34.6) | 51 (65.4) |  | Ref. |  | 45 (56.2) | 35 (43.8) |  | Ref. |  |
| Full-term |  | 87 (45.6) | 104 (54.4) | 0.573 | 1.16 | 0.69 - 198 | 69 (35.9) | 123 (64.1) | 0.837 | 0.94 | 0.54 - 1.64 | 83 (43.2) | 109 (56.8) | 0.051 | 1.69 | 0.99 - 2.86 |

|  |  | **Breastfeeding continuation to 1 year** | | | | | **Breastfeeding continuation to 2 years** | | | | |
| --- | --- | --- | --- | --- | --- | --- | --- | --- | --- | --- | --- |
|  |  | *No*  *Mean ± S.D. or N (%)* | *Yes*  *Mean ± S.D. or N (%)* | *p-value* | *OR* | *95% Confidence interval (CI)* | *No*  *Mean ± S.D. or N (%)* | *Yes*  *Mean ± S.D. or N (%)* | *p-value* | *OR* | *95% Confidence interval (CI)* |
| **Age** |  | 31.9 ± 4.7 | 31.8 ± 4.7 | 0.823 | 0.99 | 0.94 - 1.05 | 31.8 ± 4.7 | 32.3 ± 4.1 | 0.486 | 1.03 | 0.95 - 1.11 |
| **Number of children** |  | 1.6 ± 0.8 | 1.7 ± 0.7 | 0.118 | 1.31 | 0.93 - 1.84 | 1.6 ± 0.8 | 1.8 ± 0.7 | 0.4 | 1.2 | 0.78 - 1.86 |
| **Number of children under 5 years** |  | 1.3 ± 0.5 | 1.3 ± 0.5 | 0.499 | 1.19 | 0.71 - 2.01 | 1.3 ± 0.5 | 1.3 ± 0.5 | 0.914 | 0.96 | 0.47 - 1.98 |
| **Educational level** |  |  |  | 0.325 |  |  |  |  | 0.151 |  |  |
| Intermediate or less |  | 5 (62.5) | 3 (37.5) |  | Ref. |  | 7 (87.5) | 1 (12.5) |  | Ref. |  |
| Secondary and technical |  | 13 (52.0) | 12 (48.0) | 0.605 | 1.54 | 0.30 - 7.87 | 21 (84.0) | 4 (16.0) | 0.811 | 1.33 | 0.13 - 14.01 |
| University |  | 41 (41.8) | 57 (58.2) | 0.268 | 2.32 | 0.52 - 10.25 | 77 (78.6) | 21 (21.4) | 0.556 | 1.91 | 0.22 - 16.39 |
| Post-graduate |  | 51 (43.2) | 67 (56.8) | 0.298 | 2.19 | 0.49 - 9.59 | 107 (90.7) | 11 (9.3) | 0.768 | 0.72 | 0.08 - 6.39 |
| **Residence (by governorate)** |  |  |  | 0.164 |  |  |  |  | 0.226 |  |  |
| Beirut |  | 31 (50.8) | 30 (49.2) |  | Ref. |  | 51 (83.6) | 10 (16.4) |  | Ref. |  |
| Beqaa |  | 2 (12.5) | 14 (87.5) | **0.013*** | 7.23 | 1.51 - 34.57 | 15 (93.7) | 1 (6.3) | 0.322 | 0.34 | 0.04 - 2.87 |
| Mount Lebanon |  | 56 (49.1) | 58 (44.4) | 0.831 | 1.07 | 0.57 - 1.99 | 101 (88.6) | 13 (11.4) | 0.354 | 0.66 | 0.27 - 1.59 |
| North Lebanon |  | 10 (55.6) | 8 (44.4) | 0.724 | 0.83 | 0.29 - 2.38 | 16 (88.9) | 2 (11.1) | 0.586 | 0.64 | 0.13 - 3.22 |
| South Lebanon |  | 11 (27.5) | 29 (72.5) | **0.022*** | 2.72 | 1.16 - 6.42 | 29 (72.5) | 11 (27.5) | 0.183 | 1.93 | 0.73 - 5.10 |
| **Number of family members living in household** |  |  |  | **0.042*** |  |  |  |  | 0.488 |  |  |
| ≤3 |  | 58 (51.3) | 55 (48.7) |  | Ref. |  | 101 (89.4) | 12 (10.6) |  | Ref. |  |
| 4 |  | 33 (38.8) | 52 (61.2) | 0.081 | 1.66 | 0.94 - 2.94 | 67 (78.8) | 18 (21.2) | **0.044*** | 2.26 | 1.02 - 4.99 |
| 5 |  | 14 (40.0) | 21 (60.0) | 0.243 | 1.58 | 0.73 - 3.42 | 29 (82.9) | 6 (17.1) | 0.307 | 1.74 | 0.60 - 5.04 |
| ≥6 |  | 4 (28.6) | 10 (71.4) | 0.118 | 2.64 | 0.78 - 8.90 | 13 (92.9) | 1 (7.1) | 0.688 | 0.65 | 0.08 - 5.39 |
| **Number of rooms in the household** |  |  |  | 0.863 |  |  |  |  | 0.975 |  |  |
| ≤2 |  | 9 (50.0) | 9 (50.0) |  | Ref. |  | 15 (83.3) | 3 (16.7) |  | Ref. |  |
| 3 |  | 27 (41.5) | 38 (58.5) | 0.522 | 1.41 | 0.49 - 4.01 | 57 (87.7) | 8 (12.3) | 0.632 | 0.7 | 0.17 - 2.97 |
| 4 |  | 35 (43.2) | 46 (56.8) | 0.601 | 1.31 | 0.47 - 3.66 | 68 (83.9) | 13 (16.1) | 0.949 | 0.96 | 0.24 - 3.78 |
| 5 |  | 28 (45.9) | 33 (54.1) | 0.76 | 1.18 | 0.41 - 3.38 | 50 (82.0) | 11 (18.0) | 0.894 | 1.1 | 0.27 - 4.46 |
| ≥6 |  | 11 (45.8) | 13 (54.2) | 0.789 | 1.18 | 0.35 - 4.02 | 22 (91.7) | 2 (8.3) | 0.417 | 0.45 | 0.07 - 3.06 |
| **Income** |  |  |  | 0.199 |  |  |  |  | 0.101 |  |  |
| No income - <450$ |  | 22 (39.3) | 34 (60.7) |  | Ref. |  | 47 (83.9) | 9 (16.1) |  | Ref. |  |
| 450$ - 999$ |  | 14 (35.9) | 25 (64.1) | 0.738 | 1.16 | 0.49 - 2.69 | 28 (71.8) | 11 (28.2) | 0.158 | 2.05 | 0.76 - 5.56 |
| 1000$ - 2000$ |  | 28 (49.1) | 29 (50.9) | 0.293 | 0.67 | 0.32 - 1.41 | 50 (87.7) | 7 (12.3) | 0.564 | 0.73 | 0.25 -2.12 |
| >2000$ |  | 46 (47.4) | 51 (52.6) | 0.33 | 0.72 | 0.37 - 1.39 | 87 (89.7) | 10 (10.3) | 0.301 | 0.6 | 0.23 - 1.58 |
| **Year of last birth** |  |  |  | **0.001*** |  |  |  |  | **0.001*** |  |  |
| 2018 |  | 6 (75.0) | 2 (25.0) |  | Ref. |  | 7 (87.5) | 1 (12.5) |  | Ref. |  |
| 2019 |  | 6 (40.0) | 9 (60.0) | 0.122 | 4.5 | 0.67 - 30.23 | 11 (73.3) | 4 (26.7) | 0.443 | 2.55 | 0.23 - 27.71 |
| 2020 |  | 8 (27.6) | 21 (72.4) | **0.024*** | 7.88 | 1.31 - 47.43 | 21 (72.4) | 8 (27.6) | 0.392 | 2.67 | 0.28 - 25.25 |
| 2021 |  | 12 (24.0) | 38 (73.4) | **0.011*** | 9.5 | 1.69 - 53.42 | 34 (68.0) | 16 (32.0) | 0.283 | 3.29 | 0.37 - 29.08 |
| 2022 |  | 17 (26.6) | 47 (73.4) | **0.014*** | 8.3 | 1.52 - 45.12 | 60 (93.8) | 4 (6.3) | 0.521 | 0.47 | 0.05 - 4.78 |
| 2023 |  | 60 (75.0) | 20 (25.0) | 1 | 1 | 0.19 - 5.36 | 77 (96.3) | 3 (3.7) | 0.287 | 0.27 | 0.02 - 2.98 |
| 2024 |  | 1 (33.0) | 2 (66.7) | 0.224 | 6 | 0.34 - 107.42 | 2 (66.7) | 1 (33.3) | 0.441 | 3.5 | 0.14 - 84.69 |
| **Occupation** |  |  |  | 0.375 |  |  |  |  | 0.451 |  |  |
| Housewife |  | 35 (45.5) | 30 (54.5) |  | Ref. |  | 43 (78.2) | 12 (21.8) |  | Ref. |  |
| Employed/Freelancer/Contractor in a scientific or health field |  | 22 (47.8) | 24 (52.2) | 0.812 | 0.91 | 0.41 - 1.99 | 42 (91.3) | 4 (8.7) | 0.081 | 0.34 | 0.10 - 1.14 |
| Employed/Freelancer/Contractor in another field |  | 38 (44.7) | 47 (55.3) | 0.931 | 1.03 | 0.52 - 2.04 | 73 (85.9) | 12 (14.1) | 0.241 | 0.59 | 0.24 - 1.43 |
| Stopping work to take care of child |  | 19 (42.2) | 36 (57.8) | 0.746 | 1.14 | 0.52 - 2.52 | 38 (84,4) | 7 (15.6) | 0.429 | 0.66 | 0.24 - 1.85 |
| Self-employed in a scientific or health field |  | 5 (33.3) | 10 (66.7) | 0.403 | 1.67 | 0.50 - 5.52 | 13 (86.7) | 2 (13.3) | 0.471 | 0.55 | 0.11 - 2.79 |
| Student/Trainee |  | 1 (33.3) | 2 (66.7) | 0.684 | 1.67 | 0.14 - 19.48 | 3 (100.0) | 0 (0.0) | - | 1 | - |
| **Cigarette smoking** |  |  |  | 0.106 |  |  |  |  | 0.622 |  |  |
| Non-smoker |  | 84 (41.4) | 119 (58.6) |  | Ref. |  | 173 (85.2) | 30 (14.8) |  | Ref. |  |
| Previous smoker |  | 13 (56.5) | 10 (43.5) | 0.169 | 0.54 | 0.23 - 1.29 | 20 (87.0) | 3 (13.0) | 0.823 | 0.87 | 0.24 - 3.09 |
| Current smoker: ≤10 |  | 7 (58.3) | 5 (41.7) | 0.256 | 0.5 | 0.15 - 1.64 | 11 (91.7) | 1 (8.3) | 0.544 | 0.52 | 0.07 - 4.21 |
| Current smoker: ≥11 |  | 6 (54.5) | 5 (45.5) | 0.394 | 0.59 | 0.17 - 1.99 | 8 (72.7) | 3 (27.3) | 0.274 | 2.16 | 0.54 - 8.62 |
| **Waterpipe smoking** |  |  |  | 0.996 |  |  |  |  | 0.441 |  |  |
| Non-smoker |  | 63 (42.9) | 84 (57.1) |  | Ref. |  | 122 (83.0) | 25 (17.0) |  | Ref. |  |
| Previous smoker |  | 17 (51.5) | 16 (48.5) | 0.367 | 0.71 | 0.33 - 1.50 | 29 (87.9) | 4 (12.1) | 0.493 | 0.67 | 0.22 - 2.08 |
| Current smoker: <1/week |  | 15 (44.1) | 19 (55.9) | 0.867 | 0.9 | 0.26 - 3.08 | 29 (85.3) | 5 (14.7) | 0.503 | 0.49 | 0.06 - 3.99 |
| Current smoker: 1-2/week |  | 5 (45.5) | 6 (54.5) | 0.818 | 0.88 | 0.28 - 2.73 | 10 (90.9) | 1 (9.1) | 0.398 | 0.41 | 0.05 - 3.27 |
| Current smoker: 3-6/week |  | 6 (46.2) | 7 (53.8) | 0.675 | 1.31 | 0.37 - 4.68 | 12 (92.3) | 1 (7.7) | 0.503 | 0.49 | 0.06 - 3.99 |
| Current smoker: ≥7/week |  | 4 (36.4) | 7 (63.6) | 0.894 | 0.95 | 0.45 - 2.01 | 10 (90.9) | 1 (9.1) | 0.745 | 0.84 | 0.29 - 2.39 |
| **Alcohol consumption** |  |  |  | 0.106 |  |  |  |  | 0.103 |  |  |
| None |  | 72 (39.6) | 110 (60.4) |  | Ref. |  | 149 (81.9) | 33 (18.1) |  | Ref. |  |
| Previous |  | 11 (64.3.0) | 14 (35.7) | 0.671 | 0.83 | 0.36 - 1.94 | 21 (84.0) | 4 (16.0) | 0.794 | 0.86 | 0.28 - 2.67 |
| Current |  | 27 (44.0) | 15 (56.0) | **0.004*** | 0.36 | 0.18 - 0.73 | 42 (100.0) | 0 (0.0) | - | - | - |
| **Taking dietary supplements** |  |  |  |  |  |  |  |  |  |  |  |
| Iron | No | 78 (43.6) | 101 (56.4) |  | Ref. |  | 151 (84.4) | 28 (15.6) |  | Ref. |  |
|  | Yes | 32 (45.7) | 38 (54.3) | 0.760 | 0.92 | 0.53 - 1.59 | 61 (87.1) | 9 (12.9) | 0.579 | 0.79 | 0.35 - 1.78 |
| Folic acid | No | 88 (44.2) | 111 (55.8) |  | Ref. |  | 171 (85.9) | 28 (14.1) |  | Ref. |  |
|  | Yes | 22 (44.0) | 28 (56.0) | 0.978 | 1.01 | 0.54 - 1.88 | 41 (82.0) | 9 (18.0) | 0.486 | 1.34 | 0.59 - 3.06 |
| Vitamin D | No | 57 (43.5) | 74 (56.5) |  | Ref. |  | 110 (84.0) | 21 (16.0) |  | Ref. |  |
|  | Yes | 53 (44.9) | 65 (55.1) | 0.824 | 0.94 | 0.57 - 1.56 | 102 (86.4) | 16 (13.6) | 0.584 | 0.82 | 0.41 - 1.66 |
| Omega 3 | No | 94 (44.5) | 117 (55.5) |  | Ref. |  | 178 (84.4) | 33 (15.6) |  | Ref. |  |
|  | Yes | 16 (42.1) | 22 (57.9) | 0.780 | 1.1 | 0.55 - 2.22 | 34 (89.5) | 4 (10.5) | 0.418 | 0.63 | 0.21 - 1.91 |
| Calcium | No | 85 (43.4) | 111 (56.6) |  | Ref. |  | 162 (82.7) | 34 (17.3) |  | Ref. |  |
|  | Yes | 25 (47.2) | 28 (52.8) | 0.621 | 0.86 | 0.47 - 1.58 | 50 (94.3) | 3 (5.7) | **0.045*** | 0.29 | 0.08 - 0.97 |
| Multi-vitamins | No | 65 (39.4) | 100 (60.6) |  | Ref. |  | 135 (81.8) | 30 (18.2) |  | Ref. |  |
|  | Yes | 45 (53.6) | 39 (46.4) | **0.034*** | 0.56 | 0.33 - 0.96 | 77 (91.7) | 7 (8.3) | **0.044*** | 0.41 | 0.17 - 0.98 |
| **Body Mass Index before pregnancy** |  | 23.3 ± 4.2 | 23.0 ± 4.6 | 0.615 | 0.99 | 0.93 - 1.04 | 23.4 ± 4.4 | 22.0 ± 4.0 | 0.087 | 0.92 | 0.84 - 1.01 |
| **Body Mass Index during the last month of pregnancy** |  | 28.3 ± 4.6 | 27.9 ± 5.0 | 0.530 | 0.98 | 0.93 - 1.04 | 28.3 ± 4.8 | 26.9 ± 4.6 | 0.118 | 0.94 | 0.86 - 1.02 |
| **Delivery place** |  |  |  | **0.035*** |  |  |  |  | 0.405 |  |  |
| Public hospital in Lebanon |  | 0 (0.0) | 4 (100.0) |  | Ref. |  | 3 (75.0) | 1 (25.0) |  | Ref. |  |
| Private hospital in Lebanon |  | 105 (44.3) | 132 (55.7) | 0.096 | 6.29 | 0.72 - 54.63 | 202 (85.2) | 35 (14.8) | 0.576 | 0.52 |  |
| Homebirth |  | 0 (0.0) | 2 (100.0) | - | 1 | - | 1 (50.0) | 1 (50.0) | 0.547 | 3 | 0.08 - 107.45 |
| Hospital outside Lebanon |  | 5 (83.3) | 1 (16.7) | - | 1 | - | 6 (100.0) | 0 (0.0) | - | 1 | - |
| **Mode of delivery** |  |  |  |  |  |  |  |  |  |  |  |
| Cesarean section with/without general anesthesia |  | 62 (43.1) | 82 (56.9) |  | Ref. |  | 123 (85.4) | 21 (14.6) |  | Ref. |  |
| Normal Vaginal delivery with/without local anesthesia |  | 48 (45.7) | 57 (54.3) | 0.677 | 0.89 | 0.54 - 1.49 | 89 (84.8) | 16 (15.2) | 0.886 | 1.05 | 0.52 - 2.13 |
| **Pregnancy period** |  |  |  |  |  |  |  |  |  |  |  |
| Pre-term |  | 25 (35.2) | 46 (64.8.6) |  | Ref. |  | 58 (81.7) | 13 (18.3) |  | Ref. |  |
| Full-term |  | 80 (46.8) | 91 (53.2) | 0.099 | 0.62 | 0.35 - 1.09 | 149 (87.1) | 22 (12.9) | 0.275 | 0.66 | 0.31 - 1.39 |
| *** p< 0.05 indicating statistical significance** | | | | | | | | | | | |
